# Tyrosine 1–phosphorylated RNA polymerase II transcribes PROMPTs to facilitate proximal promoter pausing and induce global transcriptional repression in response to DNA damage

**DOI:** 10.1101/gr.278644.123

**Published:** 2024-02

**Authors:** Kamal Ajit, Adele Alagia, Kaspar Burger, Monika Gullerova

**Affiliations:** 1 Sir William Dunn School of Pathology, Oxford, OX1 3RE, United Kingdom;; 2 Mildred Scheel Early Career Center for Cancer Research, University Hospital Würzburg, 97080 Würzburg, Germany;; 3 Department of Biochemistry and Molecular Biology, Biocenter of the University of Würzburg, 97074 Würzburg, Germany

## Abstract

DNA damage triggers a complex transcriptional response that involves both activation and repression of gene expression. In this study, we investigated global changes in transcription in response to ionizing irradiation (IR), which induces double-strand breaks in DNA. We used mNET-seq to profile nascent transcripts bound to different phosphorylated forms of the RNA polymerase II (RNA Pol II) C-terminal domain (CTD). We found that IR leads to global transcriptional repression of protein-coding genes, accompanied by an increase in antisense transcripts near promoters, called PROMPTs, transcribed by RNA Pol II phosphorylated on tyrosine 1 (Y1P) residue of the CTD. These Y1P-transcribed PROMPTs are enriched for PRC2 binding sites and associated with RNA Pol II proximal promoter pausing. We show the interaction between Y1P RNA Pol II and PRC2, as well as PRC2 binding to PROMPTs. Inhibition of PROMPTs or depletion of PRC2 leads to loss of transcriptional repression. Our results reveal a novel function of Y1P-dependent PROMPTs in mediating PRC2 recruitment to chromatin and RNA Pol II promoter pausing in response to DNA damage.

RNA polymerase II (RNA Pol II) is an essential enzyme that catalyzes the transcription of protein-coding genes (PCGs) and noncoding regulatory RNA species, such as microRNAs, small nuclear RNAs, and snoRNAs (Cramer et al. 2008). The RNA Pol II activity is a complex process that requires its interaction with multiple regulatory proteins. The largest subunit of RNA Pol II, RPB1, contains an evolutionarily conserved carboxyl-terminal domain known as CTD (Liu et al. 2008). This domain consists of 52 heptapeptide repeats, Tyr1–Ser2–Pro3–Thr4–Ser5–Pro6–Ser7, in mammals. The CTD repeats can be phosphorylated or glycosylated on Tyr1, Ser2, Thr4, Ser5, and Ser7 residues. Dynamic phosphorylation of CTD residues enables the recruitment of different RNA Pol II interacting proteins that are crucial for regulation of transcription initiation, elongation, and termination, as well as a number of cotranscriptional processes (Komarnitsky et al. 2000).

Phosphorylation of serine 5 (S5P) of RNA Pol II peaks near the promoter region and gradually diminishes as it extends toward the 3′ terminus of the gene. S5P is intimately associated with the initiation of transcription and the subsequent release of paused RNA Pol II from the promoter region (Liu et al. 2004). In contrast, serine 2 phosphorylation (S2P) commences at the promoter region and increases in density toward the 3′ end of a gene. S2P plays a pivotal role in facilitating transcription elongation through the recruitment of various kinases (Heidemann et al. 2013). Additionally, S2P facilitates interactions between RNA Pol II, splicing factors, and transcription termination factors toward the 3′ end of a gene (Lunde et al. 2010; David et al. 2011). Tyrosine 1 phosphorylation (Y1P) marks are predominantly located in proximity to promoters and enhancers of PCGs (Descostes et al. 2014). Mutation of CTD Y1 residues to nonphosphorylatable phenylalanines can lead to transcription termination defects and, subsequently, result in increased transcriptional readthrough in mammals (Shah et al. 2018). Studies have also reported a connection between Y1P and antisense transcription occurring a few hundred base pairs upstream of the transcription start site (TSS) in mammals (Descostes et al. 2014). More recently, Y1P has been shown to play a crucial role in the generation of damage-responsive transcripts (DARTs) in the vicinity of double-strand breaks (DSBs), functioning as a binding platform for DNA repair proteins (Burger et al. 2019). Even though the role of Y1P has been intricately linked with the maintenance of genomic integrity in response to DNA damage, the specific contribution of Y1P-mediated antisense transcription near the promoter region to the broader context of the DNA damage response (DDR) remains unexplored.

DNA is constantly exposed to harmful radiation and chemical agents that can induce various forms of damage, resulting in single-strand breaks (SSBs) and DBSs. The repair of DSBs is orchestrated by two main DDR pathways: homologous repair (HR) and nonhomologous end joining (NHEJ). HR, which is a precise repair mechanism, requires a homologous sister chromatid as a template and predominantly occurs in the S/G_2_ phase of cell cycle. In contrast, NHEJ is more error-prone but functions throughout interphase. Importantly, the efficiency of both HR and NHEJ can be enhanced by active transcription near DSBs (Burger et al. 2019).

In general, transcription of genes in the vicinity of DSBs is suppressed through the initial action of the ATM serine/threonine kinase (ATM) (Iannelli et al. 2017), an upstream signaling molecule in the DDR pathway. Transcriptional down-regulation has been shown to correlate with the distance from DSBs. Furthermore, when ATM was inhibited, the repression of gene expression is impaired, highlighting the active regulation of this process by DDR signaling pathways (Iannelli et al. 2017).

A mechanism for global transcriptional shutdown through degradation of ubiquitinated RNA Pol II has been shown upon UV-induced DNA damage. However, UV induces DNA SSBs, activating repair pathways such as base excision repair (BER), nucleotide excision repair, and mismatch repair, which are different from DSB-induced HR or NHEJ (Rastogi et al. 2010). Therefore, a mechanism for global transcriptional response could be distinct from UV-induced transcriptional repression and remains enigmatic.

The pausing of newly recruited RNA Pol II at the promoter is a critical step of transcription regulation in mammals (Gressel et al. 2019). RNA Pol II pausing occurs within a 200-bp region downstream from the TSS, where it accumulates until the positive elongation factor B (P-TEFb) triggers the release of the paused RNA Pol II, allowing progression into the transcription elongation stage. This transition is also marked by a change in the CTD modification, transitioning from S5P to S2P. Chromatin remodeling, such as chromatin condensation triggered by trimethylation of lysine 27 on histone 3 (H3K27me3), is associated with RNA Pol II proximal promoter pausing. Recently, the Polycomb repressive complex II (PRC2) has been shown to act as a master regulator of transcriptional repression through RNA Pol II proximal promoter pausing in mouse embryonic stem cells (Rosenberg et al. 2021). PRC2 binds to nascent transcripts near the promoter region, especially to antisense transcripts located a few hundred base pairs upstream of the TSS, known as promoter upstream transcripts (PROMPTs) (Kanhere et al. 2010). The recruitment of PRC2 by PROMPTs depends on the presence of specific GC-rich motifs (Rosenberg et al. 2021). Once bound, PRC2 then drives RNA Pol II pausing through chromatin condensation. Although PRC2 has been linked to DSB repair (Campbell et al. 2013), its role in transcriptional repression during ionizing irradiation (IR)-induced DSBs remains unknown.

Mammalian nascent elongating transcript sequencing (mNET-seq) can be used to effectively examine the levels of nascent transcripts and identify the location of actively transcribing RNA Pol II. One of the major advantages of mNET-seq is that it provides a snapshot of actively transcribing RNA Pol II positions through mapping of the free 3′ OH on the reads still attached to the polymerase. A further advantage of mNET-seq is the use of different antibody species, such as Y1P, S2P, and S5 RNA Pol II, to pull down and sequence transcripts bound to RNA Pol II at various stages of transcription. Here, we used mNET-seq to investigate global transcriptional repression following IR and to elucidate the mechanisms behind it.

## Results

### IR-induced DNA damage leads to global down-regulation of PCGs

**Figure 1.**
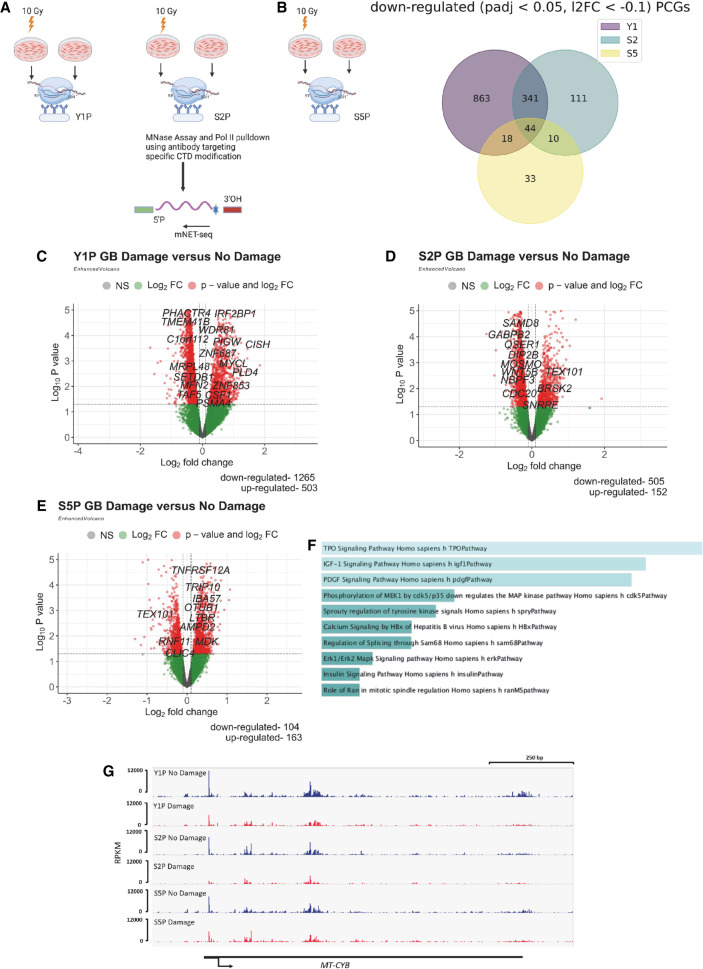
IR-induced DNA damage leads to global down-regulation of protein-coding genes (PCGs). (*A*) mNET-seq experimental design shows the sample preparation of irradiated and nonirradiated U2OS cells from which Y1P, S2P, and S5P RNA Pol II were precipitated for mNET-seq. (*B*) Venn diagram of significantly down-regulated (*P*adj < 0.05, log_2_FC < −0.1) PCGs based on read coverage across the gene body (GB) from Y1P, S2P, and S5P samples after IR. (*C*) Volcano plots of differentially expressed PCGs based on read coverage across GB from Y1P samples upon IR. (*D*) Volcano plots of differentially expressed PCGs based on read coverage across GB from S2P samples upon IR. (*E*) Volcano plots of differentially expressed PCGs based on read coverage across GB from S5P samples upon IR. (*F*) GO enrichment of significantly down-regulated genes across Y1P, S2P, and S5P samples upon IR. (*G*) IGV profile of mNET-seq signal across a representative down-regulated gene, *MT-CYB*.

Although several studies have reported that transcription of genes in proximity to DSBs is transiently repressed, the genome-wide transcriptional response to DSBs has not been previously investigated. Here, we used mNET-seq to study global nascent transcriptomic changes upon IR. To elucidate how differently modified CTD of RNA Pol II facilitates transcriptomic changes during IR, we sequenced transcripts bound to Y1P, S2P, and S5P CTD of RNA Pol II (Fig. 1A). We used coverage of reads across the gene body (GB) (200 bp downstream from the annotated TSS to the TES) to measure expression of a gene. This ensures that reads from paused RNA Pol II that do not contribute to active transcription in the proximal promoter region (50 bp upstream of to 200 bp downstream from the TSS) are excluded from downstream differential expression analysis. Principal component analysis (PCA) of three biological replicates (Supplemental Fig. S1A–C) showed 35% variation along PC2 between the irradiated and nonirradiated samples. Differential expression analysis was then performed for Y1P, S2P, and S5P between the irradiated and nonirradiated samples with DESeq2. Overall, we detected that there are more PCGs that were significantly down-regulated (log_2_FoldChange [log_2_FC] < −0.1, *P*adj < 0.05) compared with significantly up-regulated genes (log_2_FC > 0.1, *P*adj < 0.05) in irradiated samples (Fig. 1B; Supplemental Fig. S2A). This was most profound for Y1P and S2P RNA Pol II (1285 down-regulated genes vs. 503 up-regulated genes and 506 down-regulated genes vs. 154 up-regulated genes, respectively) (Fig. 1C,D). In contrast to Y1P and S2P, there were more up-regulated genes associated with S5P RNA Pol II in irradiated samples (Fig. 1E). Pathway enrichment analyses of these up-regulated genes (Supplemental Fig. S2B) showed enrichment for TP53 signaling and cell cycle regulation, which are crucial for transcription of DDR genes and cell cycle arrest (Liu and Kulesz-Martin 2001). Specifically, we found *CDKN1A* (Cazzalini et al. 2010) among the up-regulated genes in irradiated samples (Supplemental Fig. S2C).

In addition, analysis of down-regulated genes (Fig. 1F) showed enrichment in various pathways such as insulin signaling, MAPK, and PDGF signaling, indicating a global repression of cellular programs linked to cell proliferation. Furthermore, we detected down-regulation of mitochondrial genes involved in oxidative phosphorylation, like *MT-CYB* (Fig. 1G) and *MT-ND5*, potentially indicating a state of reduced cell survival.

Conversely, differential analysis of long noncoding RNA (lncRNA) showed an opposite trend when compared with PCGs. We detected predominantly up-regulated expression of lncRNA upon IR, specifically for transcripts associated with Y1P RNA Pol II (Supplemental Fig. S3A,B). Furthermore, 60.4% of up-regulated Y1P lncRNAs are natural antisense transcripts that could trigger transcriptional interference of sense genes (Supplemental Fig. S3C; Modarresi et al. 2012). Nascent lncRNA transcripts associated with S2P RNA Pol II were mostly down-regulated (Supplemental Fig. S3D,E), whereas S5P transcription of lncRNA resulted in few detectable changes upon IR (Supplemental Fig. S3F).

Overall, our data showed that IR-induced DNA damage leads to general down-regulation of PCGs and up-regulation of lncRNA, in particular, those that are in antisense orientation and associated with Y1P RNA Pol II.

### DNA damage results in increased expression of antisense PROMPTs

**Figure 2.**
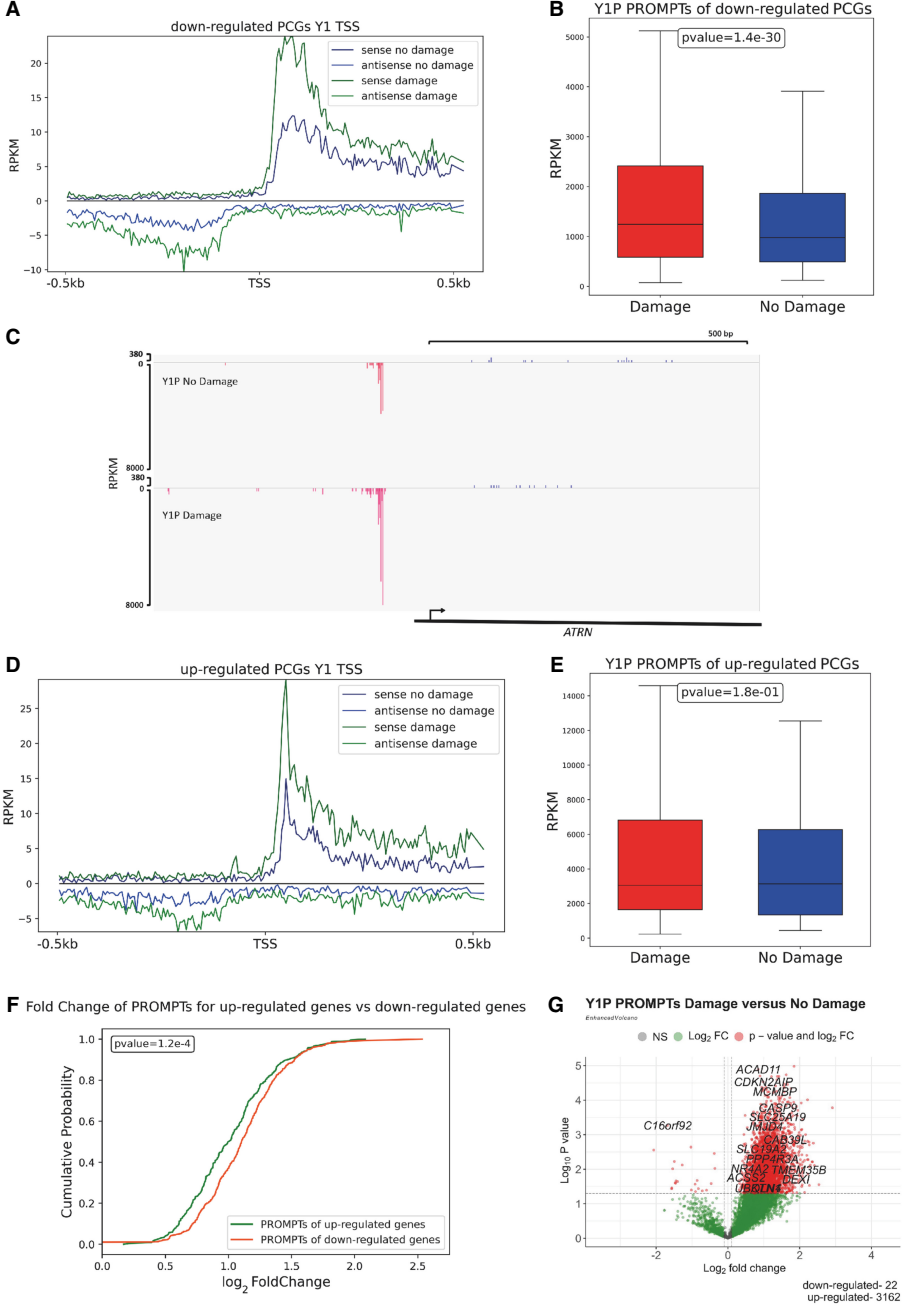
DNA damage results in increased expression of antisense promoter upstream transcripts (PROMPTs). (*A*) mNET-seq metagene profiles showing sense and antisense transcription near the TSS of down-regulated PCGs from Y1P-damage and no-damage samples. (*B*) Box plot indicates mNET-seq read coverage across PROMPTs of down-regulated PCGs from Y1P-damage and no-damage samples. A two-sample Wilcoxon test is used to compute statistical significance of difference in PROMPT expression for down-regulated genes between the Y1P-damage and Y1P-nondamage samples. (*C*) IGV profile of mNET-seq signal across a representative down-regulated gene showing increased PROMPT expression. (*D*) mNET-seq metagene profiles showing sense and antisense transcription near the TSS of up-regulated PCGs from Y1P-damage and no-damage samples. (*E*) Box plot indicates mNET-seq read coverage across PROMPTs of up-regulated PCGs from Y1P-damage and no-damage samples. (*F*) Cumulative distribution plot showing the log_2_FC of PROMPTs upon IR between up-regulated and down-regulated PCGs from Y1P samples. The Mann–Whitney *U* test is used for statistically testing the medians between the log_2_FC of PROMPT distribution between up-regulated and down-regulated PCGs upon IR from Y1P samples. (*G*) Volcano plots of differentially expressed PROMPTs from Y1P upon IR.

Our data indicate that IR results in up-regulation of nascent antisense lncRNA associated with Y1P RNA Pol II. To further investigate the role of antisense lncRNA up-regulation upon IR, we analyzed the antisense reads 500 bp upstream of the TSS of PCGs. These regions can initiate the production of antisense PROMPTs and modulate the transcription of a downstream PCG (Chellini et al. 2020). To interrogate the changes in PROMPT expression, we used mNET-seq metagene profiles showing their reads around the annotated TSS of PCGs. mNET-seq average profiles showed a global, significant increase in Y1P-associated PROMPTs upon IR (Supplemental Fig. S4A,B). Furthermore, down-regulated PCGs associated with Y1P showed a significant (*P*-value < 0.05) increase in PROMPT expression (Fig. 2A–C; Supplemental Fig. S4C) upon IR compared with up-regulated PCGs (Fig. 2D,E; Supplemental Fig. S4D). Finally, a cumulative distribution plot (Fig. 2F) confirmed a significant (*P-*value = 1 × 10^−4^) increase in log_2_FC in PROMPT expression for PROMPTs associated with down-regulated genes compared with up-regulated PCGs.

Next, we performed a differential expression analysis of reads mapping to PROMPT-corresponding regions. We detected a significant number (3162) of up-regulated Y1P-associated PROMPTs upon IR (*P-*value < 0.05, log_2_FC > 0.1) compared with 22 significantly down-regulated (*P-*value < 0.05, log_2_FC < −0.1) Y1P-associated PROMPTs (Fig. 2G). These results show that Y1P-associated PROMPTs are up-regulated upon IR and that they are significantly more up-regulated around the TSS of transcriptionally down-regulated PCGs. We also detected, in line with Y1P, up-regulation of PROMPTs associated with S2P (Supplemental Fig. S4E,F). Previous studies in nondamage conditions showed that PROMPTs are generally transcribed by Y1P RNA Pol II, whereas S2P is generally associated with the GB (Descostes et al. 2014). Therefore, it is possible that the observations for S2P could be attributed to overlap in the type of RNA Pol II precipitated and cross-specificity between antibodies.

### Y1P RNA Pol II–transcribed PROMPTs are associated with an increased proximal promoter pausing index upon IR

Proximal promoter pausing has been shown to be the critical rate-limiting step in determining a gene's transcriptional output (Gressel et al. 2019). Nascent RNA-seq techniques like mNET-seq, which determines the position of actively transcribing RNA polymerases, can be used to calculate the promoter pause index (PI) (Szlachta et al. 2018). Promoter PI is defined as the ratio of RNA Pol II density across the proximal promoter region to the density of RNA Pol II across the GB (Day et al. 2016). A higher PI is indicative of greater RNA Pol II pausing.

**Figure 3.**
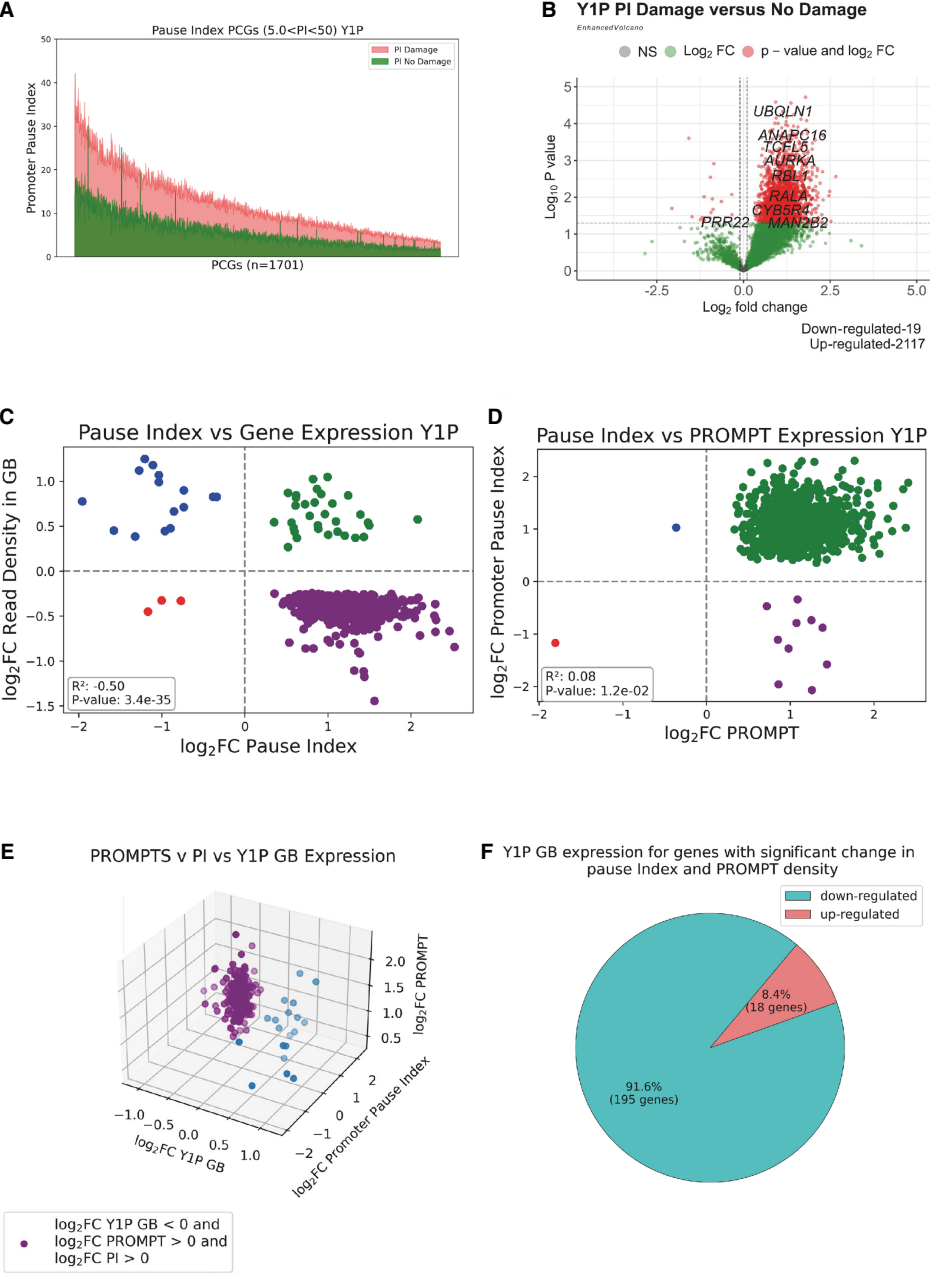
Y1P RNA Pol II–transcribed PROMPTs are associated with an increased promoter pausing index upon IR. (*A*) Line plot showing change in the pause index (PI) of PCGs from Y1P samples upon IR. Genes with a moderate PI (5 < PI < 50) are sorted based on the PI, and each point on the *x*-axis represents a gene; green lines show its PI in a no-damage condition, and orange lines show its PI in a damage condition. (*B*) Volcano plots showing differential PI of PCGs from Y1P samples upon IR. (*C*) Scatter plot showing the log_2_FC in GB expression with the associated log_2_FC in PI of PCGs from Y1P samples on radiation treatment. Only PCGs that show a significant *P*-value (*P* < 0.05) change in the PI and GB from Y1P upon IR were included in the analysis. (*D*) Scatter plot showing the log_2_FC in PROMPT expression with the associated log_2_FC in the PI of PCGs from the Y1P sample on radiation treatment. Pearson correlation coefficient and *P*-value show correlation between PROMPT expression and the PI change of PCGs from Y1P sample on radiation treatment. Only PCGs that show a significant *P*-value (*P* < 0.05) change in the PROMPT expression and PI from Y1P samples upon IR were included in the analysis. (*E*) Three-dimensional scatter plot comparing the log_2_FC in GB expression with the log_2_FC in PI and log_2_FC in the PROMPT of PCGs from Y1P samples on radiation treatment. Only PCGs that show a significant (*P* < 0.05) change in PROMPT expression, PI, and GB from Y1P samples upon IR were included in the analysis. (*F*) Pie chart showing the percentage of down-regulated and up-regulated genes among the PCGs that show a significant (*P* < 0.05) change in PROMPT expression, PI, and GB from Y1P samples upon IR.

To investigate if there is a correlation between RNA Pol II pausing and transcriptional repression, we compared the PI in both IR and non-IR conditions and showed that almost all the genes with a significant change in the PI have a significantly (*P-*value < 0.05) increased PI upon IR (Fig. 3A; Supplemental Fig. S5A,B). This observation is also corroborated by differential expression analysis using the PI, confirming that almost all genes that showed significant increase in PI were also up-regulated (Fig. 3B). Overall, we observed a significantly increased PI in Y1P samples upon IR. Increased PI values have been also observed for S2P and S5P RNA Pol II (Supplemental Fig. S5C–G), suggesting that this is a general trend following DNA damage induction. GO enrichment analysis of the Y1P-transcribed genes with significantly increased PI revealed enrichment for double-strand DNA repair pathways (Supplemental Fig. S6A). However, the read coverage of DNA repair pathway genes across the GB (Supplemental Fig. S6B) was significantly increased, despite elevated read coverage across the proximal promoter region of these genes (Supplemental Fig. S6C). Hence, it is possible that greater recruitment of RNA Pol II dominates over RNA Pol II pausing to secure expression of DDR repair genes upon IR.

Overall, we observed a significantly increased PI in Y1P samples upon IR. Moreover, 94% (476 out of 509) of genes that show significant changes in PI were significantly down-regulated in their GB (Fig. 3C). Furthermore, 99.9% (951 out of 952) of genes that showed significant changes in the PI were associated with significant up-regulation in their PROMPT expression (Fig. 3D). All together, we observed a significant correlation between increases in PI and Y1P PROMPT expression and down-regulation of expression in the GB (Fig. 3E). Specifically, 92% of Y1P significantly down-regulated genes were associated with a significant increase in PROMPT expression and PI (Fig. 3F). We also detected, similarly to Y1P, although at lower levels, up-regulation of S2P PROMPTs and PI upon IR (Supplemental Fig. S7A,B). Up-regulated PI positively correlated with up-regulated PROMPTs and down-regulation of expression in the GB (Supplemental Fig. S7C,D). Similarly, 92.6% of S2P PCGs that show a significant change in GB expression, Y1P PROMPT expression, and associated PI were significantly down-regulated in GB expression along with a significant increase in Y1P PROMPT expression and PI (Supplemental Fig. S7E,F). This illustrates that transcription of PROMPTs is linked to decreased expression of the associated gene via pausing. S5P transcription also showed a generally similar trend, however, at much lower levels (Supplemental Fig. S8A–D). In summary, we detected a correlation between down-regulation of gene expression with concomitant increase in the PI and PROMPT expression. Hence, up-regulated PROMPTs can be linked to transcriptional repression of PCGs through pausing of RNA Pol II in response to DNA damage.

### IR-induced Y1P-transcribed PROMPTs are enriched for PRC2 binding motifs

We have shown that a significant increase in PROMPT expression is correlated with a greater pausing of RNA Pol II, leading to possible global transcriptional repression. Prior studies have indicated that antisense transcription near the promoter region could modulate gene expression of an upstream gene (Chellini et al. 2020). One of the key mechanisms through which PROMPTs can repress gene expression is by recruiting PRC2 (Rosenberg et al. 2021). PRC2 promotes chromatin condensation through trimethylation of histone H3K27, leading to RNA Pol II pausing (Kanhere et al. 2010). PRC2 has been shown to have a binding preference for four-stranded G-rich RNA structures (Fay et al. 2017; Kwok and Merrick 2017), known as G-quadruplex (G4) structures (Wang et al. 2017), at proximal promoter regions.

**Figure 4.**
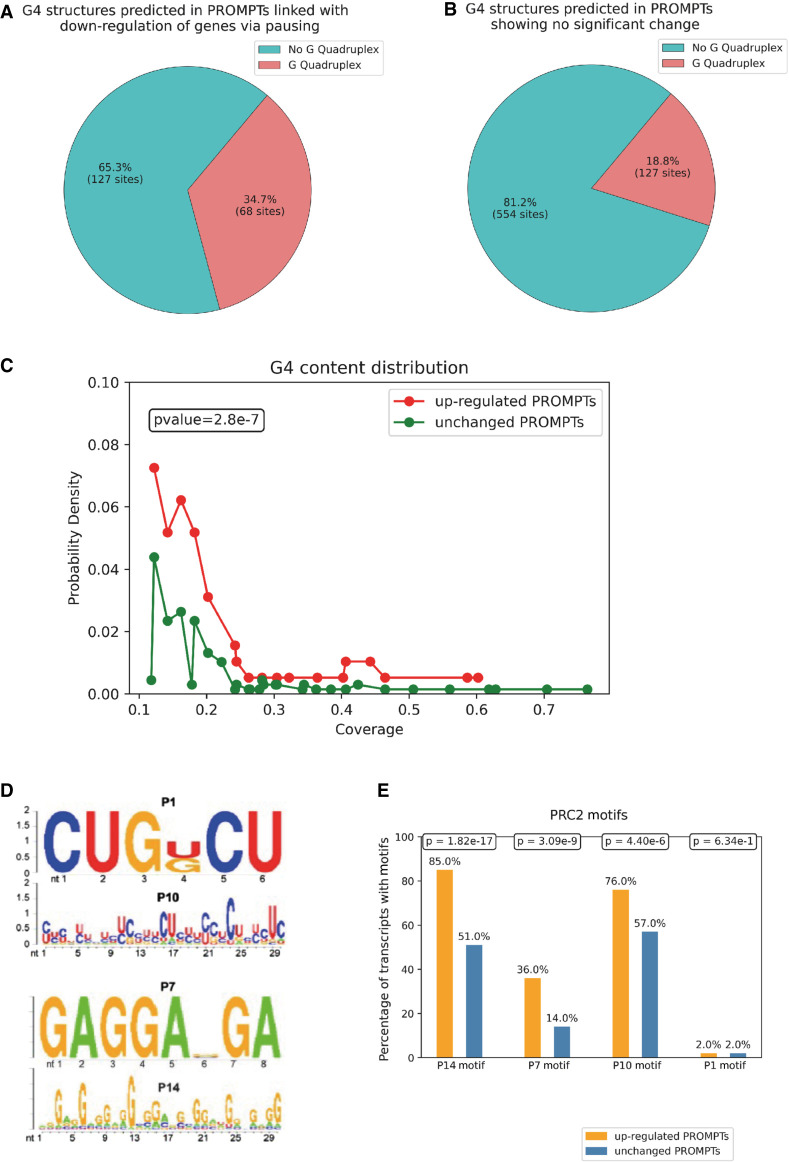
IR-induced Y1P-transcribed PROMPTs are enriched in PRC2 binding motifs. (*A*) Pie chart showing the proportion of sequences predicted to harbor a rG4 structure among the up-regulated (*P-*value < 0.05, log_2_FC > 0.1) PROMPTs, which are correlated with down-regulated GB expression and increased PI upon IR from Y1P samples. (*B*) Pie chart showing the proportion of sequences predicted to harbor a rG4 structure among the unchanged (−0.05 log_2_FC < 0.05 or *P*-value > 0.05) PROMPTs upon IR from Y1P. (*C*) Probability density plots representing rG4 coverage across up-regulated PROMPTs, which are linked with down-regulated GB expression and increased PI versus unchanged PROMPTs upon IR from Y1P samples. A Mann–Whitney *U* test is used to compare median values of the two probability distributions. (*D*) RNA-binding motifs of PRC2 identified using dCLIP (Rosenberg et al. 2021). (*E*) Bar charts showing the percentage of sequences containing a PRC2 motif among the up-regulated (*P-*value < 0.05, log_2_FC > 0.1) PROMPTs, which are correlated with down-regulated GB expression and increased PI upon IR from Y1P samples versus unchanged PROMPTs. A Mann–Whitney *U* test was to compute statistical significance in the difference in enrichment of PRC2 motifs between up-regulated and unchanged PROMPTs.

To investigate the presence of G4 motifs within PROMPTs, we analyzed IR-induced Y1P-associated PROMPTs using a deep learning–based prediction tool, G4RNA screener. We detected a higher enrichment (34.8% compared with 18.8%) of G4 motifs in the up-regulated Y1P PROMPTs, which are linked to gene down-regulation, compared with PROMPTs that are not increased (*P*-value −0.05 < log_2_FC < 0.05) upon IR (Fig. 4A,B). Using probability density plots, we showed a significant increase in G4 coverage in the up-regulated PROMPTs linked with gene down-regulation via PI compared with unchanged PROMPTs (Fig. 4C).

A previous study identified four different PRC2 binding motifs (Fig. 4D; Rosenberg et al. 2021) by sequencing RNA bound to PRC2 using dCLIP assays (Rosenberg et al. 2017). We observed, similar to the G4 enrichment, significantly higher enrichment for the PRC2 binding motifs P14 (*P-*value = 1.82 × 10^−17^), P7 (*P-*value = 3.09 × 10^−9^), and P10 (*P-*value = 4.40 × 10^−6^) in up-regulated PROMPTs linked to gene down-regulation via RNA Pol II pausing (Fig. 4E). The U-rich P1 motif did not show a difference in enrichment between the two groups of PROMPTs, suggesting that GC-rich motifs (P10, P7, and P14) likely form scaffolds for PRC2 binding.

Apart from PRC2 binding motifs, up-regulated PROMPTs also showed enrichment for motifs of splicing factors like RBM4 and SRSF7 (Supplemental Fig. S9), which were shown to be correlated with the proximal promoter pausing in a previous study (Akcan et al. 2023). The exact mechanisms for the action of these splicing factors on transcription repression remains enigmatic.

All together, our data suggest that Y1P PRMOPTs that are up-regulated in response to DNA damage are G4-rich and contain PRC2 binding motifs.

### PRC2 is in close proximity to Y1P RNA Pol II and PROMPTs upon DNA damage

Because IR-up-regulated Y1P-associated PROMPTs contain PRC2 binding motifs, we hypothesized that PRC2 and Y1P RNA Pol II might be in close proximity in the cells. Human PRC2 consists of four core subunits (enhancer of zeste 2 polycomb repressive complex 2 subunit [EZH2], embryonic ectoderm development [EED], SUZ12 polycomb repressive complex 2 subunit [SUZ12], and RB binding protein 7/4, chromatin remodeling factor (RBBP7/RBBP4; also known as RbAp46/RbAp48) and several auxiliary subunits. EZH2 is known as the enzymatic subunit of PRC2 (Shi et al. 2017).

**Figure 5.**
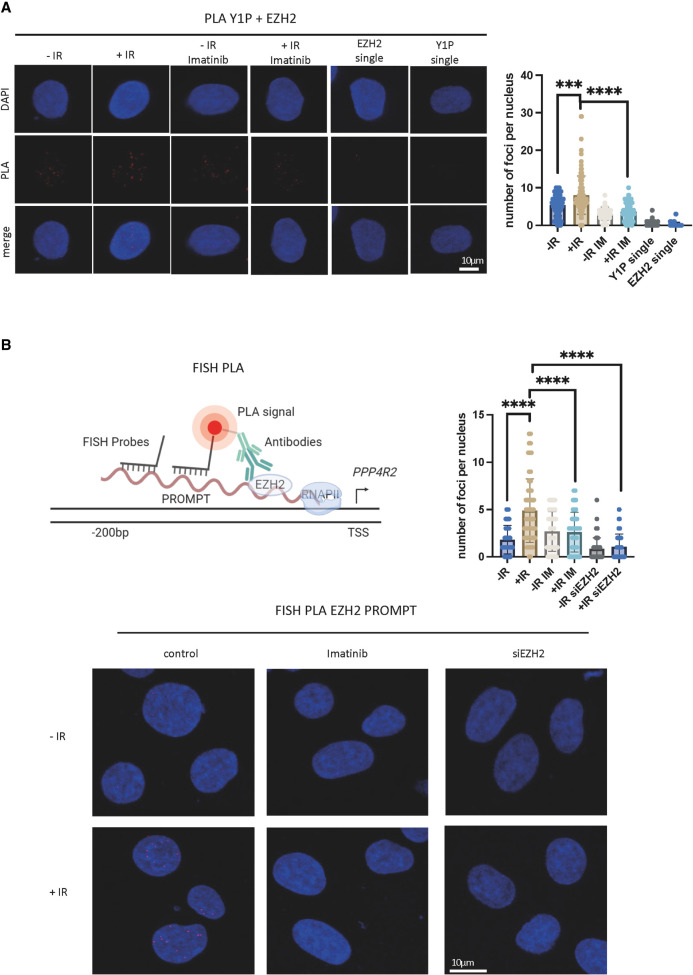
PRC2 is in close proximity to Y1P RNAPII and PROMPTs upon DNA damage. (*A*, *left*) Representative images showing PLA foci of Y1P and EZH2 in presence or absence of imatinib (1 μM, 1 h) in IR (10 Gy, 10 min) or no-IR conditions. n(no IR) = 126, n(IR) = 113, n(no IR + imatinib) = 104, n(IR imatinib) = 75, n(EZH2 single) = 41, and n(Y1P single) = 52, where n denotes the number of nuclei used for quantification. (*Right*) Quantification of PLA. A Mann–Whitney *U* test was used to compute statistical significance for difference in foci count. (*) *P* < 0.05, (**) *P* < 0.01, (***) *P* < 0.001, and (****) *P* < 0.0001. (*B*, *top left*) Illustration showing principles of FISH-PLA. (*Bottom*) Representative images showing FISH-PLA foci in presence or absence of imatinib (1 μM, 1 h) in IR (10 Gy, 10 min) or no-IR conditions or in wt or EZH2-depleted cells. (*Top right*) Quantification of FISH-PLA images. A Mann–Whitney *U* test was used to compute statistical significance for difference in foci count. (****) *P* < 0.0001.

To evaluate the proximity of PRC2 and Y1P RNA Pol II, we performed a proximity ligation assay (PLA) using antibodies against EZH2 (Duan et al. 2020), and Y1P. We detected a significantly (*P-*value < 0.05) increased number of PLA foci, suggesting interaction between EZH2 and Y1P RNA Pol II upon DSB induction (single antibodies were used as negative control) (Fig. 5A). We have shown previously that damage-induced ABL1 (also known as c-ABL) is the kinase responsible for phosphorylating Y1 CTD on RNA Pol II at DSBs. The activity of ABL1 can be inhibited by imatinib treatment. Treatment with imatinib resulted in a significant decrease in PLA foci compared with the untreated sample upon IR (Fig. 5A). These data suggest that Y1P RNA Pol II is in close proximity to PRC2.

To investigate whether PRC2 can directly bind to PROMPTs, we used fluorescent in situ hybridization in combination with PLA (FISH-PLA) (Alagia et al. 2023). In this assay, instead of using two antibodies (as in traditional PLA detecting interaction between two proteins of interest), we use one antibody (against EZH2) and oligonucleotide probes specific to PROMPTs (on one selected gene from mNET-seq). Analysis of FISH-PLA revealed a significant, IR-induced interaction between EZH2 and PROMPTs, which was sensitive to imatinib treatment and depletion of EZH2 (Fig. 5B; Supplemental Fig. S11A).

Next, we tested whether EZH2 binding to PROMPTs is caused by its increased expression upon IR treatment. We tested two different cell lines, U2OS and HEK293, and used western blot analysis to show that IR does not lead to reproducible up-regulation of EZH2 protein levels (Supplemental Fig. S10A). Because EZH2 protein levels remain similar between irradiated and nonirradiated samples, the increased interaction is most likely owing to enhanced expression of PROMPTs.

### PROMPTs and PRC2 facilitate down-regulation of PCGs upon DNA damage

**Figure 6.**
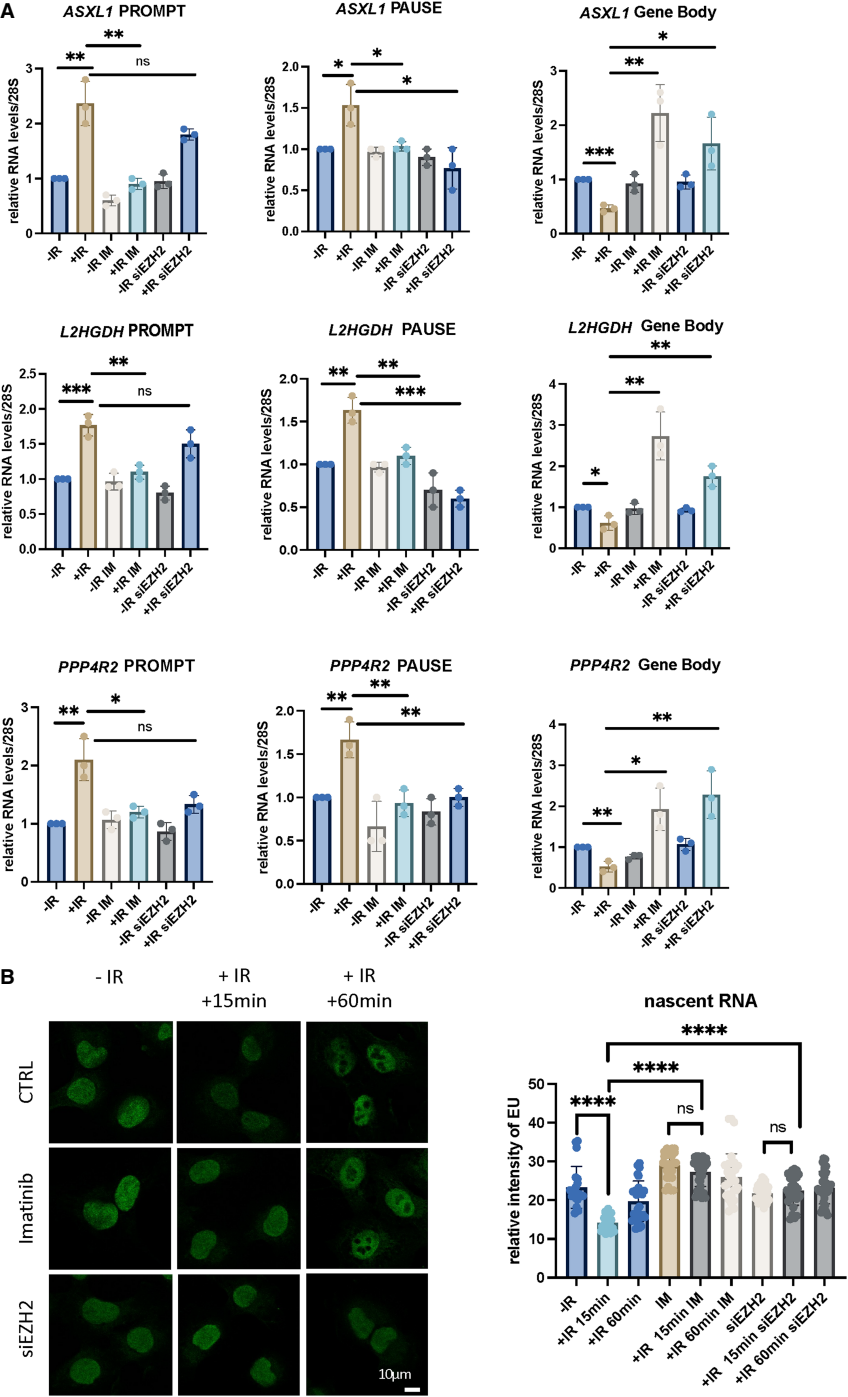
PROMPTs and PRC2 facilitate down-regulation of PCGs upon DNA damage. (*A*) Bar charts showing RT-qPCR analysis of PROMPT-, PAUSE-, and GB-derived RNA at three selected genes. An unpaired *t*-test was used to assess statistical significance. (*) *P* < 0.05, (**) *P* < 0.01, (***) *P* < 0.001. (*B*, *left*) Representative images showing EU staining of nascent RNA in cells exposed to IR. (*Right*) A bar chart showing the quantification of images on *left*. A Mann–Whitney *U* test was used to compute statistical significance for difference in foci count: (****) *P* < 0.0001.

If indeed Y1P PROMPTs serve as a recruitment platform for PRC2, which in turn leads to proximal promoter pausing and down-regulation of an upstream gene, inhibition of PROMPTs or depletion of EZH2 should have an effect on the targeted gene expression. To test this hypothesis, we performed RT-qPCR analysis using three strand-specific primer sets: one probing for antisense PROMPTs, one probing for sense promoter pausing–derived RNA, and one probing for sense RNA in the body of the gene (Supplemental Fig. S10B). RT-qPCR was performed on RNA isolated from wild-type (WT), imatinib-treated, or EZH2-depleted cells exposed to IR. We selected three representative genes from our mNET-seq analysis, *ASXL1*, *L2HGDH*, and *PPP4R2*, that were significantly down-regulated upon DNA damage and associated with elevated Y1P PROMPT expression. Our RT-qPCR data revealed that IR induced an increase in antisense PROMPTs and sense pause-derived RNA alongside a decrease in RNA levels in the GB. The IR-induced increase in PROMPT RNA was greater and more significant than the increase in 5′ pause-derived RNA. Furthermore, treatment with imatinib or depletion of EZH2 abolished the observed IR-induced gene repression (Fig. 6A). To test whether this damage-induced transcription repression is also present in another noncancer cell line, we performed RT-qPCR in HEK293 cells and obtained similar results, confirming PROMPT-driven transcription repression is a general mechanism (Supplemental Fig. S10C).

Next, we used EU staining of nascent RNA followed by CLICK chemistry and confocal microscopy to show global down-regulation of nascent transcription upon IR. This transcriptional repression was sensitive to imatinib treatment and EZH2 depletion (Fig. 6B; Supplemental Fig. S11B).

To examine whether up-regulation of PROMPTs is a phenomenon also observed during SSBs induced by UV, we examined nascent RNA from GRO-seq data corresponding to significantly differentially expressed genes 8 h after UV exposure (Williamson et al. 2017). We did not observe a significant increase in PROMPT expression for down-regulated genes (Supplemental Fig. S12A–D) following UV exposure. This might be because of the ephemeral nature of PROMPTs, limiting their effects to a short duration (<30 min) after DNA damage. To test PROMPTs at a shorter time after UV exposure (30 min), we used comprehensive RT-qPCR analysis of three selected genes that are regulated by this mechanism upon IR treatment. We did not detect down-regulation of these genes or increased expression of PROMPTs upon UV treatment (Supplemental Fig. S12E).

Overall, our data indicate that up-regulation of PROMPTs for transcriptionally repressed genes might be unique to IR-induced DNA damage.

## Discussion

DNA damage often entails a need for transcriptional repression of genes near the damage sites to prevent defective and obstructive transcription until the DNA damage is repaired. Prior studies using UV-induced DNA damage have suggested that global transcriptional shutdown is stimulated by the degradation of ubiquitinated RNA Pol II during SSB repair (Tufegdžić Vidakovic et al. 2020; Steurer et al. 2022). Studies using the sequence-specific AsiSI cell line model showed ATM-dependent transcriptional down-regulation of genes near cleavage sites. However, the molecular principles that control the global transcriptional response to IR-induced DSBs remain enigmatic. In this study, we used mNET-seq strategy to provide evidence for global transcriptional repression of PCGs following IR. We observed that the down-regulated pathways included a myriad of growth-related factors and other signaling pathways, whereas up-regulated genes were involved in TP53 signaling and DNA repair pathways, implying a shift in transcriptome expression from normal cell division and growth to DNA repair and cell cycle arrest. Additionally, we detected an increase in the expression of numerous lncRNA species, specifically antisense RNA transcribed by Y1P RNA Pol II following IR. This represents a sharp dichotomy to the repressed transcriptome of PCGs. The antisense lncRNA could modulate gene expression of PCGs in *cis* via transcriptional interference (Zhao et al. 2020), whereby transcription of antisense RNA can interfere with RNA Pol II transcribing the overlapping sense PCG. We show that natural antisense transcripts, Y1P-transcribed PROMPTs, were significantly up-regulated and associated with a subset of down-regulated PCGs following IR.

Increased Y1P PROMPT expression in response to DNA damage has not been reported before. Previously, we showed that Y1P facilitates transcription of DARTs at DSBs (Burger et al. 2019). The activity of Y1P RNA Pol II at DSBs is regulated and triggered by damage-induced ABL1 kinase, which phosphorylates Y1 in the CTD of RNA Pol II. Up-regulation of ABL1 was also reported as a consequence of activation of upstream DDR regulators such as DNA-dependent protein kinase (DNA-PK) and ATM. Another study (Yurko et al. 2017) showed overall increased levels of Y1P following IR in *Saccharomyces cerevisiae*. We show a significant increase in the density of Y1P RNA Pol II in the antisense region, 500 bp upstream of the TSS, represented in irradiated samples. However, a question remains as to how Y1P activity is regulated and preferentially associated with promoter regions of down-regulated genes. Perhaps differences in sequence or epigenetic modifications might be relevant to determine the preference for antisense transcription at a subset of genes upon IR.

The increased expression of Y1P PROMPTs upstream of down-regulated genes was found to be positively correlated with RNA Pol II proximal promoter pausing. A previous study (Iannelli et al. 2017) on transcriptional repression close to DSBs in an AsiSI model cell line also reported a decrease in transcription initiation using CAGE sequencing, which quantifies the 5′ end of nascent RNA of genes near DSBs. This study also observed a significant reduction in transcription elongation by quantifying S2P using ChIP-seq. Indeed, reduced transcription elongation is consistent with increased proximal promoter pausing, which prevents the transition from S5P to S2P (Liu et al. 2015). Based on the GB coverage of S2P from mNET-seq, the increased number of significantly down-regulated PCGs compared with significantly up-regulated genes (505 vs. 152) further supports the reduction in transcription elongation reported during DSB induction. Furthermore, our data suggest that the reduction in transcription elongation is a global phenomenon in response to IR-induced DSBs.

PROMPTs have been shown to modulate the transcription of upstream PCGs (Chellini et al. 2020). In particular, transcription repression through the recruitment of PRC2 (Kanhere et al. 2010) can condense chromatin through H3K27me3 (Rosenberg et al. 2021). PRC2 recruited by PROMPTs can also induce RNA Pol II proximal promoter pausing through catalytic inactivation of a transcription elongation factor known as elongin A (ELOA) (Ardehali et al. 2017). The significant enrichment of PRC2 binding motifs in Y1P PROMPTs associated with gene down-regulation through pausing, as well as our FISH-PLA data, suggests Polycomb-dependent transcriptional repression upon IR. Furthermore, using RT-qPCR and EU staining, we showed recovery of nascent RNA synthesis upon PRC2 knockdown in irradiated cells. Suppressing PROMPT expression by reducing Y1P RNA Pol II also restored nascent RNA synthesis in response to DNA damage, suggesting that Y1P-transcribed PROMPTs mediate transcription repression in a PRC2-dependent manner. The role of Polycomb complexes in DDR was reported previously. PRC1-mediated and ATM-dependent transcription repression of genes near DSBs was previously shown using a U2OS cell line with I-SceI restriction sites inducing DSBs at known sites (Ui et al. 2015). The study also showed that recruitment of PRC1 through ATM induces histone H2A monoubiquitination, which can impede transcription elongation (Zhou et al. 2008). However, PRC1 can also be recruited to H3K27me3 marks deposited by PRC2 (Laugesen et al. 2019). Hence, it is likely that PRC1-mediated transcriptional repression might be one of the complementary effects of PRC2 recruitment to Y1P PROMPTs. Additionally, PRC2 alone can also lead to RNA Pol II pausing via H3K27me3-induced chromatin condensation.

Because PROMPTs are transcribed by Y1P RNA Pol II, which is dependent on elevated levels of ABL1 kinase upon DDR, we also checked for ABL1 expression across cancer types to ascertain whether our proposed mechanism is a unique feature of osteosarcoma cells (bone cancer). The elevated expression of ABL1 across several cancer types compared with noncancerous counterparts (Supplemental Fig. S13) suggests that PROMPT-mediated transcriptional repression upon DSBs might be conducive in a variety of cancers. However, in cancers of the immune system like acute lymphoblastic leukemia, myelodysplastic malignancies are characterized by loss of function mutation in EZH2 (Ernst et al. 2010; Chase et al. 2020), and therefore would not be able to facilitate PRC2-dependent transcription repression owing to an inability to catalyze H3K27me3 repressive chromatin marks.

**Figure 7.**
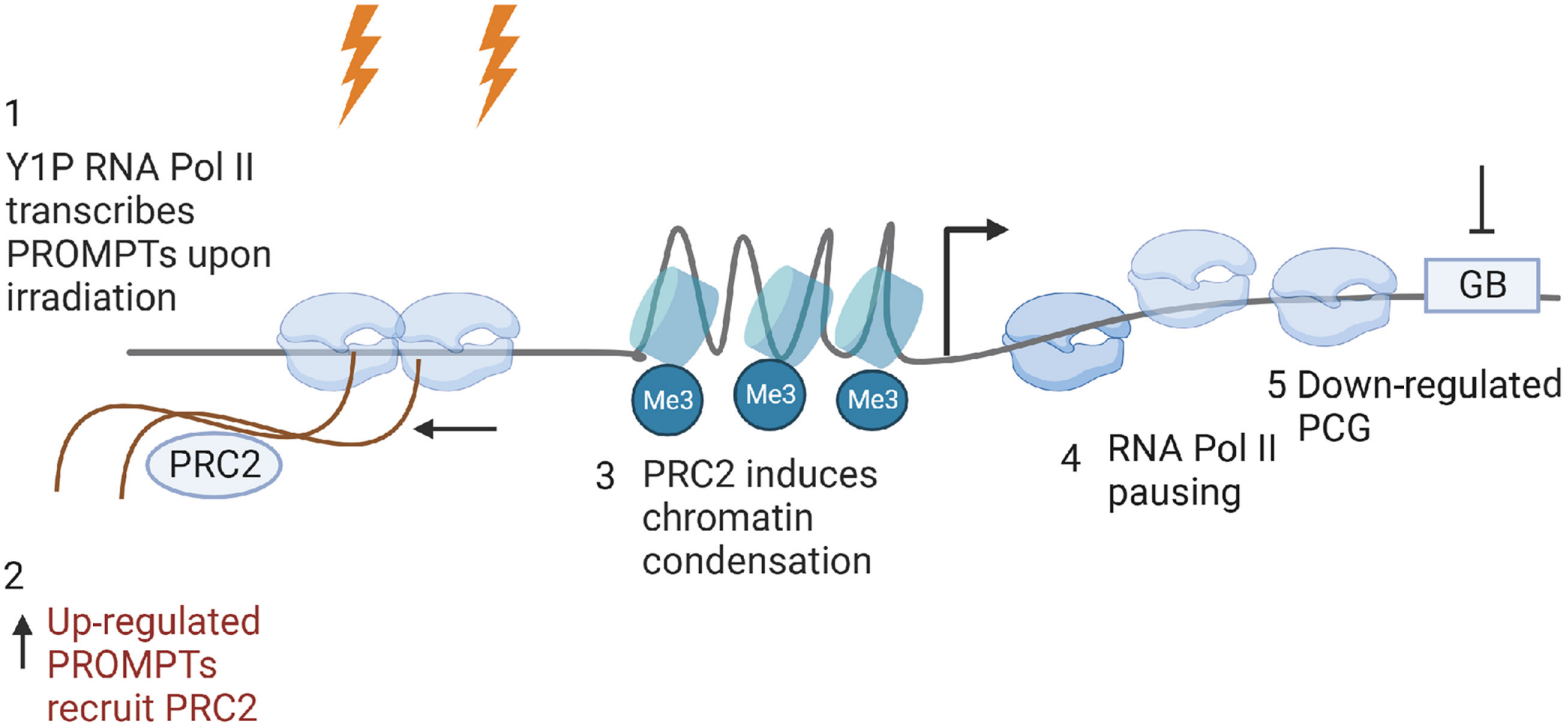
Model of Y1P RNAPII-dependent PROMPTs facilitating down-regulation of PCGs upon DNA damage. Upon DSB induction, elevated ATM-dependent ABL1 activity causes greater enrichment of Y1P RNA Pol II near the promoter of PCGs and concomitantly enhanced expression of PROMPTs near PCGs. PROMPTs recruit PRC2, which facilitates pausing of RNA Pol II via H3K27me3-dependent chromatin condensation, leading to reduced gene expression of PCGs. Image was created with BioRender (https://www.biorender.com).

In conclusion, we used mNET-seq to illustrate genome-wide transcriptional repression of PCGs upon DSB induction. We propose that following IR, Y1P-transcribed PROMPTs form a recruitment platform for the transcriptional repressor complex, PRC2, which in turn deposits a repressive mark on histones, leading to RNA Pol II stalling near the TSS and, consequently, resulting in the down-regulation of the downstream gene (Fig. 7). This transcriptional repression might contribute to efficient DNA repair.

## Methods

### Cell culture

U2OS cells were obtained from ATCC and maintained in monolayer culture at exponential growth in high-glucose Dulbecco's Modified Eagle Medium supplemented with 10% heat-inactivated fetal bovine serum and 1× penicillin/streptomycin solution. Cells were incubated at 37°C in a humidified environment with 5% CO_2_ and periodically checked for the presence of mycoplasma contamination. To induce DNA damage, cells were irradiated with 10 Gy, and samples were collected 30 min after treatment.

### Proximity ligation assay

The PLA was executed following the guidelines provided by the Duolink in situ red or green starter kit for mouse/rabbit (Sigma-Aldrich), adhering to the manufacturer's instructions. Cells were seeded onto coverslips followed by fixing and permeabilization according to established immunofluorescence protocol. Permeabilization of the cells was performed using CSK buffers (10 mM PIPES, 100 mM NaCl, 300 mM sucrose, 3 mM MgCl_2_, 1 mM EGTA, 0.7% Triton X-100, 1 × protease inhibitor cocktail) in a cold room for 15 min before fixation. Subsequently, the coverslips were subjected to a 1-h blocking step in PLA blocking buffer within a humidity chamber maintained at 37°C. Primary antibodies were diluted using PLA antibody diluent and applied to the coverslips, which were then incubated overnight at 4°C in a humidified chamber. Following this incubation, the coverslips were washed three times in PLA buffer A at room temperature before being exposed to PLA probes for 1 h at 37°C. After another three washes in PLA buffer A, the coverslips were treated with PLA ligase in ligation buffer for 30 min at 37°C. Subsequent to three washes in PLA buffer A, the coverslips were incubated with PLA polymerase in polymerase buffer for 100 min at 37°C in the absence of light. Postincubation, the coverslips were washed three times in PLA buffer B under dark conditions and then rinsed twice with 0.01× PLA buffer B. Finally, the coverslips were mounted onto slides using PLA DAPI solution and imaged using an Olympus Fluoview FV1200 confocal microscope equipped with a 60× objective. For image visualization, Fiji (Schindelin et al. 2012) was used, and subsequent analysis was performed using CellProfiler (Carpenter et al. 2006) with an in-house pipeline.

### FISH-PLA

FISH-PLA was performed following the guidelines previously outlined (Alagia et al. 2023). U2OS cells were initially seeded onto glass coverslips and, after 24 h, were subjected to IR (10 Gy) and incubated for 10 min. The coverslips were then washed thrice with cold PBS and fixed in cold 4% paraformaldehyde (Alfa Aesar) in PBS for 15 min. Following three additional washes in cold PBS, permeabilization was performed with cold 0.5% Triton X-100 in PBS (PBST) for 10 min. Slides were then subjected to three washes in cold PBS and blocked in a buffer consisting of 1× SSC, 20 μg/μL yeast tRNA (Thermo Fisher Scientific), 20 μg/μL salmon sperm DNA (Thermo Fisher Scientific), 0.5% Triton X-100, 2% BSA, and 1 U/μL RNasin plus (Promega) for 1 h at 37°C.

The probe incubation buffer, comprising 1× SSC and 100 nM DNA probes, underwent heating for 3 min to 98°C, followed by cooling on ice for 10 min before the addition of 1% Triton X-100 and 1 U/μL RNasin plus (final). Coverslips were then incubated with the probe incubation buffer for 3 min at 80°C and, subsequently, overnight at 37°C. Postincubation, coverslips were washed three times in SSC-T buffer (1× SSC, 0.1% Triton X-100, 1% BSA) and twice in PBS before blocking for 1 h at 37°C with PLA blocking buffer. Primary antibodies were appropriately diluted in PLA antibody diluent and incubated overnight at 4°C in a humidified chamber. Cover slips were then washed three times in PLA buffer A at room temperature, followed by incubation with minus rabbit PLA probe (Sigma-Aldrich) for 1 h at 37°C. The PLA protocol was then carefully followed according to the manufacturer's instructions using the Duolink in situ red starter kit mouse/rabbit (Sigma-Aldrich).

### Western blot

Protein samples were extracted from cell culture using RIPA buffer. They were then sonicated for a duration of 10–30 sec, followed by a 5-min incubation at 98°C in 1× Laemmli buffer (Alfa Aesar). Subsequently, these treated samples were loaded onto 4%–15% Mini-PROTEAN TGX gels (Bio-Rad) and electrophoresed in Tris-glycine running buffer (Bio-Rad) under a constant voltage of 100 V for a period of 1 h. For the purpose of transferring proteins onto nitrocellulose membranes, gels were transferred over a span of 1 h 30 min at a consistent voltage of 15 V. The successful transference of proteins to the membranes was assessed using Ponceau S staining (Merck). The nitrocellulose membranes were then subjected to blocking, which was performed for 1 h at room temperature using 5% milk in PBS with 0.1% Triton X-100 (PBST). Following blocking, the membranes were incubated overnight at 4°C with primary antibodies. After primary antibody incubation, the membranes underwent three PBST washes and were subsequently incubated with secondary antibodies for 1 h at room temperature. After an additional three PBST washes, protein bands were visualized using an enhanced chemiluminescence (ECL) detection, and the results were recorded on Amersham Hyperfilm ECL film.

### RNA transfection

U2OS cells were reverse-transfected using 60 nM of siRNA targeting EZH2 and Lipofectamine RNAiMAx following manufacturer's instructions. Twenty-four hours after transfection, cells were irradiated with 10 Gy and harvested after 15 min.

### Isolation of RNA and RT-qPCR

Total RNA was isolated from U2OS cells using TRIzol reagent, treated with DNase I following the manufacturer's instructions, and quantified by NanoDrop. A reverse transcription reaction was performed using gene-specific primers and SuperScript III enzyme according to the manufacturer's instructions. The reference gene 28S was used as an internal control. All primer pairs were purchased from Sigma-Aldrich, and Primer BLAST was used as primer designing tool.

### 5-EU labeling of cultured cells and detection by click chemistry

U2OS cells were seeded on glass coverslips to reach 70% of confluency after 24 h. 5-EU was added to complete the culture medium to a final concentration of 2 mM for 1 h. Before IR, cells were washed twice with 1× phosphate buffer saline solution followed by incubation with complete culture media. Irradiated cells (10 Gy) were harvested at specific time points (15 min and 1 h); nonirradiated cells were used as negative control. The fixation and permeabilization steps were performed using PFA 4% solution for 10 min at 37°C and Triton 0.5% solution for 10 min at room temperature. Click reaction was performed for 1 h at room temperature protected from light using CuSO_4_ 2 mM, THPTA 10 mM, aminoguanidine 5 mM, sodium ascorbate 100 mM, and biotin-PEG_3_-azide 100 µM solution in PBS; all reagents were prepared fresh and used within 30 min from resuspension. The reaction was quenched by washing the cells for three times (5-min rocking at room temperature) with a solution of Triton X-100 0.5% and EDTA 5 mM in PBS. Then, cells were blocked with BSA 5% and Triton X-100 0.1% solution for 1 h at 37°C. Antibiotin and γH2AX primary antibodies (1:1000 dilution) were incubated overnight at 4°C in BSA 5% and Triton X-100 0.1%. Secondary antibody incubation (1:2000 dilution) was performed for 2 h at room temperature and protected from light using Alexa Fluor 555 goat antirabbit and Alexa Fluor 488 goat antimouse. Cells were counterstained with DAPI and imaged on an Olympus FV1200 live cell confocal microscope.

### mNET-seq sample preparation

mNET-IP samples were prepared as described previously (Burger et al. 2019). Specifically, 4 μg of relevant antibody was coupled to magnetic beads (Invitrogen) overnight, washed, and resuspended in 100 μL NET-2 buffer (50 mM Tris–HCl at pH 7.4, 150 mM NaCl, 0.05% NP-40) before immunoprecipitation. Irradiated and control cells were harvested, washed in cold PBS, and lysed in hypotonic lysis buffer (10 mM HEPES at pH 7.9, 60 mM KCl, 1.5 mM MgCl_2_, 1 mM EDTA, 1 mM DTT, 0.075% NP-40, 1× protease/phosphatase inhibitor cocktails, Roche; 10 min, 4°C with rotation). Nuclei were collected by centrifugation (2 min, 1000 rpm, 4°C), washed 2× in hypotonic lysis buffer without NP-40, and resuspended in 125 μL cold NUN1 buffer (20 mM Tris–HCl at pH 7.9, 75 mM NaCl, 0.5 mM EDTA, 50% glycerol, 1× protease/phosphatase inhibitor cocktails, Roche). Then, 1.2 mL NUN2 buffer (20 mM HEPES-KOH at pH 7.6, 300 mM NaCl, 0.2 mM EDTA, 7.5 mM MgCl_2_, 1% NP-40, 1 M urea, 1× protease/phosphatase inhibitor cocktails, Roche) was added, and nuclei were incubated on ice (15 min) with occasional vortexing and centrifuged (10 min, 16,000 rpm, 4°C). The nonsoluble chromatin pellet was washed in 100 μL 1× MNase buffer (NEB), centrifuged, and digested in 100 μL MNase reaction mix (87 μL ddH_2_O, 10 μL 10× MNase buffer, 1 μL 100× BSA, 2 μL MNase [2000 U/μL] for 90 sec at 37°C with rotation). Ten microliters of 100 mM EDTA was added to stop digestion. MNase digests were centrifuged (5 min, 16,000 rpm, 4°C), and the supernatant was diluted with 10 volumes of NET-2 buffer. Conjugated antibodies were added to the diluted supernatants and incubated for 2 h at 4°C with rotation. mNET-IP was performed in absence of Empigen. Samples were centrifuged (5 min, 2000 rpm, 4°C), and pelleted beads were washed in 800 μL NET-2 buffer 7×. RNA was recovered using TRIzol, resuspended in 20 μL urea dye (7 M urea, 0.05% xylene cyanol, 0.05% bromophenol blue), incubated for 10 min at 75°C, and separated (30 min, 350 V) in 1× TBE buffer (90 mM Tris, 90 mM boric acid, 2 mM EDTA) on a 6% PAGE gel with 7 M urea. Migration fronts of xylene cyanol and bromophenol blue or end-labeled pBR322 MspI digest (NEB) were used as size marker. The separated RNA was size-selected into a small (<100-nt) fraction by cutting out gel slices according to size markers. Slices were incubated in 400 μL elution buffer (1 M NaOAc, 1 mM EDTA; 2 h, room temperature with rotation). Samples were centrifuged (2 min, 13,000 rpm), and supernatants containing eluted RNA were loaded on spin-X-columns (Coster) and centrifuged (1 min, 13,000 rpm). Flow-through was precipitated using 1 ml 100% ethanol and 1 μL glycogen (MP Bio), incubated (20 min, room temperature), and centrifuged (20 min, 13,000 rpm). Pellets containing small RNA were resuspended in 6 μL ddH_2_O. RNA quality was controlled using a Bioanalyzer (Agilent).

### mNET-seq data processing

mNET-seq data were processed following mNET-seq processing pipeline (Prudêncio et al. 2020). In brief, adapters were trimmed using cutadapt (version 4.4) (Martin 2011; https://cutadapt.readthedocs.io/en/stable/installation.html) in paired-end mode, and the quality of the resulting FASTQ files was assessed using FASTQC (https://www.bioinformatics.babraham.ac.uk/projects/FASTQc/). The trimmed reads were then aligned to the human hg38 reference genome using STAR aligner (Dobin et al. 2013). Uniquely mapped reads were then extracted from the alignment files using SAMtools (version 1.7) (Li et al. 2009). Reads resulting from PCR internal priming and duplication events were removed using the Python script (https://github.com/kennyrebelo/Filtering_InternalPriming) from Prudêncio et al. (2020). The resulting BAM files were then used for differential gene expression analysis. To make mNET-seq plots representing locations of actively transcribing RNA Pol II, the free 3′OH end of RNA fragments was obtained using the script at GitHub (https://github.com/rluis/mNET_snr) (Prudêncio et al. 2020). Free 3′OH ends of reads emanating from intronic lariats or splicing intermediates that might occlude the position of RNA Pol II were removed by removing reads mapping to ends of exons using SAMtools (version 1.7) and BEDTools (version 2.31.0) (Quinlan and Hall 2010). The final alignment files containing positions of free three 3′OH were then used for making gene plots and Integrative Genomics Viewer (IGV) profiles.

### Metagene plots

Coverage files containing RPKM normalized read counts per nucleotide position were generated for each alignment using deepTools BAMCoverage (https://deepTools.readthedocs.io/en/develop/). The coverage files were exported in bigWig format and loaded into the IGV browser (https://software.broadinstitute.org/software/igv/) for visualizing the normalized read density around break sites. Subsequently, computeMatrix operation of deepTools was used to calculate the average profile from the bigWig files with bin size set at five. The scale region mode of computeMatrix was used to make metagene plots of GB regions with 1-kb flanks. The reference point mode was used to make metagene plots around the TSS with 500-bp flanks upstream of and downstream from the TSS.

### Differential expression analysis

For differential expression analysis, alignment files containing full read length after removal of internal priming and duplication events were used.

To measure gene expression, GB was defined as a region starting from 200 bp downstream from the TSS to the TES in order to exclude paused RNA Pol II near the promoter region. Read counts across the GB were calculated using featureCounts: (https://www.rdocumentation.org/packages/Rsubread/versions/1.22.2/topics/featureCounts) with parameters set to exclude reads that overlap gene bodies, multimapping reads, and reads that are bound to the opposite strand of the annotated feature. DESeq2 (Love et al. 2014) was then used to perform differential expression analysis. For differential expression, the number of replicates was set to four, and the batch effect was corrected for in the design matrix by attributing the technical replicate as the same batch. Significantly up-regulated genes were defined as adj *P*-value (Mann–Whitney *U* test after correcting for multiple testing) < 0.05 and log_2_FC > 0.1, and significantly down-regulated adj *P*-value < 0.05 and log_2_FC > 0.1. Volcano plots were created using the EnhancedVolcano (https://bioconductor.org/packages/devel/bioc/vignettes/EnhancedVolcano/inst/doc/EnhancedVolcano.html) package of R (R Core Team 2021). Gene Ontology analysis for DEGs was performed using Enrichr (Kuleshov et al. 2016).

### PROMPT analysis

PROMPTs were defined as transcripts in the region 500 bp upstream of the annotated TSS in antisense direction. For differential expression analysis of PROMPTs, antisense read coverage in the 500 bp upstream of the TSS was obtained using BEDTools multicov. The BED file containing co-ordinates for 500 bp upstream of the TSS in antisense direction was obtained by using a custom Python script to modify BED entries of hg38 reference genome. The PROMPT read counts were normalized based on total number of reads in each sample replicate. PROMPTs with a zero read count in any of the samples were removed from analysis, and a *P*-value of 0.05 resulting from a Mann–Whitney *U* test was set as the significance threshold for differential expression. A custom Python script was used to calculate log_2_ (average PROMPT expression of no-damage replicates/average PROMPT expression of damage replicates) and to perform statistical testing using the Python SciPy package.

Cumulative distribution plots were made using a custom Python script to compare fold changes in PROMPT expression for up-regulated genes versus down-regulated genes based on their GB expression. Statistical testing was performed for this plot with a Mann–Whitney *U* test from the SciPy Python package.

Total read coverage in the 500-bp region in the antisense direction was obtained using BEDTools multicov for up-regulated and down-regulated genes based on GB expression in damage and no-damage samples. Box plots were then made with Matplotlib and significance testing performed with a two-sample Wilcoxon test to compare PROMPT expression in damage and no-damage samples for up-regulated and down-regulated genes based on GB expression.

### PI analysis

The promoter PI was defined as the ratio between read coverage across the promoter-proximal region (PPR) and GB region. PPR was defined as 50 bp upstream of and 200 bp downstream from the annotated TSS. GB was defined as the region from 50 bp downstream from the annotated TSS to the annotated TES. The PPR was defined arbitrarily based on the peak of the paused RNA Pol II from metagene plots. The read coverages across PPR and GB were determined using BEDTools muticov. The read coverages across PPR and GB were normalized based on sample depth. Genes with zero read counts in any of the samples for either PPR and GB were removed, along with genes that have GB <1000 bp. A Mann–Whitney *U* test was used to test the significant change in PI between the control and condition. A custom Python script was used to calculate log_2_ (average Pi expression of condition replicates/average pi expression of condition replicates) and then subsequently presented as a volcano plot using EnchancedVolcano R package.

Genes that show a significant change (*P* < 0.05) in PI between the condition and control were also plotted in the same graph as two curves using Matplotlib. Matplotlib was also leveraged to represent genes that show a significant change in PI, PROMPT expression, and GB expression as a 3D scatter plot.

### PRC2 motif enrichment analysis

The RNA-binding motif PRC2 as position weight matrices were obtained from Rosenberg et al. (2021). SEA (https://meme-suite.org/meme/tools/sea) was then used to interrogate the significant enrichment of PRC2 binding sites in significantly up-regulated PROMPTs linked with gene down-regulation via pausing compared with PROMPTs that do not show significant change in expression (−0.05 < log_2_FC < 0.05) upon IR. SEA also provides statistical testing to show if there is significant enrichment of binding motifs in the input set of sequences compared with the background. The subsequent output from SEA was plotted as a bar chart and the *P*-value displayed using the Matplotlib Python package. SEA was also used with its built-in RNA-binding protein database to analyze differential enrichment of all known RBP motifs on PROMPTs.

### rG4 structure analysis

rG4-containing subsequences within PROMPTs were predicted using G4RNA screener (Garant et al. 2017). The percentage coverage of rG4 sequences within PORMPTs was calculated using a Python script. Probability density plots representing rG4 coverage and percentage of PROMPTs with that rG4 coverage were plotted using Matplotlib for significantly up-regulated PROMPTs linked with gene down-regulation via pausing and unchanged (−0.05 <log_2_FC < 0.05) PROMPTs. Statistical significance of change in rG4 coverage between the two sets of PROMPTs were evaluated using Mann–Whitney *U* test using SciPy Python package.

### GRO-seq analysis

GRO-seq reads corresponding to UV-irradiated samples, 8 h after UV exposure, and untreated samples were obtained from the NCBI Gene Expression Omnibus (GEO; https://www.ncbi.nlm.nih.gov/geo/) under the accession number GSE91012 (Williamson et al. 2017). Adapters were then automatically detected and trimmed using Trim Galore! (https://github.com/FelixKrueger/TrimGalore). The trimmed reads were then aligned to the human hg38 reference genome using STAR aligner. Uniquely mapped reads were then extracted from the alignment files using SAMtools (version 1.7) and split into forward- and reverse-stranded reads. A set of significantly down-regulated (*P*adj < 0.05, log_2_FC < −0.5) and significantly up-regulated (*P*adj < 0.05, log_2_FC > 0.5) PCGs from RNA-seq data comparing untreated samples with UV exposed samples, 8 h after exposure, were obtained from the Supplemental Data of Williamson et al. (2017). The PROMPT coverage from GRO-seq alignment files was then calculated for down-regulated and up-regulated genes using BEDTools multicov. Metagene plots were also made using GRO-seq alignment files for the set of up-regulated and down-regulated genes.

### Statistics

Foci numbers from PLA were quantified by cell profiler software. For laser, fluorescence intensity was measured by software ImageJ. The Mann–Whitney *U* test was performed using GraphPad Prism (https://www.graphpad.com/features) to check for statistical significance. An asterisk denotes *P* < 0.05; two asterisks, *P* < 0.01; three asterisks, *P* < 0.001; and four asterisks, *P* < 0.0001. The Mann–Whitney *U* test was used pervasively for all experiments except for comparing PROMPT expression between up-regulated and down-regulated genes, which are based on two-sample Wilcoxon testing.

Details about reagents used in this study can be found in Supplemental Tables 1 and 2.

## Data access

All raw and unprocessed mNET-seq data generated in this study have been submitted to the NCBI Gene Expression Omnibus (GEO; https://www.ncbi.nlm.nih.gov/geo/) under accession number GSE244353. Code is provided as Supplemental Code.

## Supplementary Material

Supplement 1

Supplement 2

Supplement 3

Supplement 4

Supplement 5

Supplement 6

Supplement 7

Supplement 8

Supplement 9

Supplement 10

Supplement 11

Supplement 12

Supplement 13

Supplement 14

Supplement 15

Supplement 16
