# Supplementary material for: Tyrosine 1–phosphorylated RNA polymerase II transcribes PROMPTs to facilitate proximal promoter pausing and induce global transcriptional repression in response to DNA damage
Source: Genome Res. 2024 Feb;34(2):201–16. doi: 10.1101/gr.278644.123 (PMC10984383; doi:10.1101/gr.278644.123)
Supplement: Supplement 1 [file Supplemental_Fig_S1.pdf]

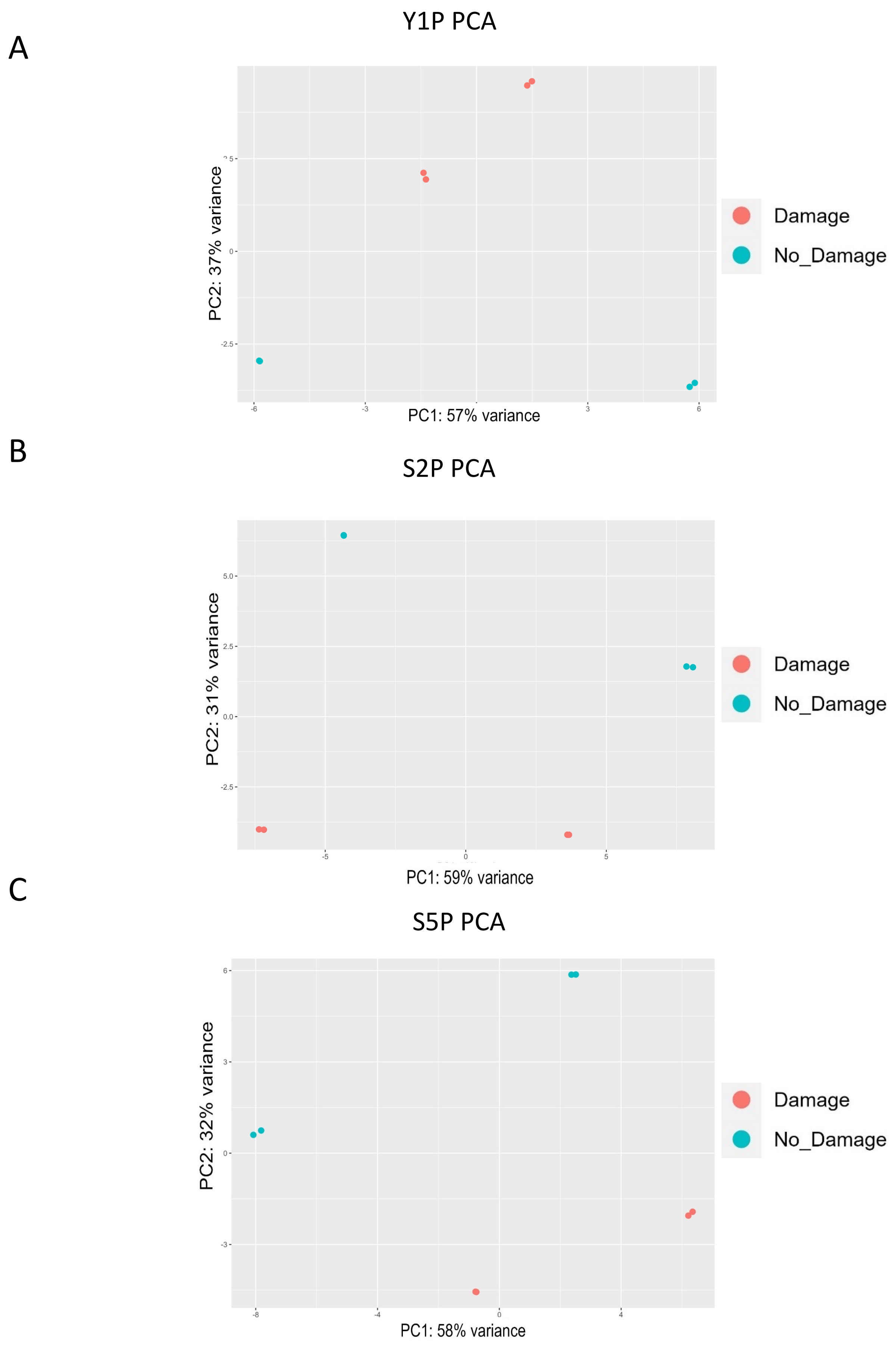

Figure S1. Principal Component Analysis of mNET-Seq read coverage across gene body. **A)** PCA plot of Y1P replicates from damage and no damage conditions**. B)** PCA plot of S2P replicates from damage and no damage conditions. **C)** PCA plot of S5P replicates from damage and no damage conditions.
