# Supplementary material for: Tyrosine 1–phosphorylated RNA polymerase II transcribes PROMPTs to facilitate proximal promoter pausing and induce global transcriptional repression in response to DNA damage
Source: Genome Res. 2024 Feb;34(2):201–16. doi: 10.1101/gr.278644.123 (PMC10984383; doi:10.1101/gr.278644.123)
Supplement: Supplement 2 [file Supplemental_Fig_S2.pdf]

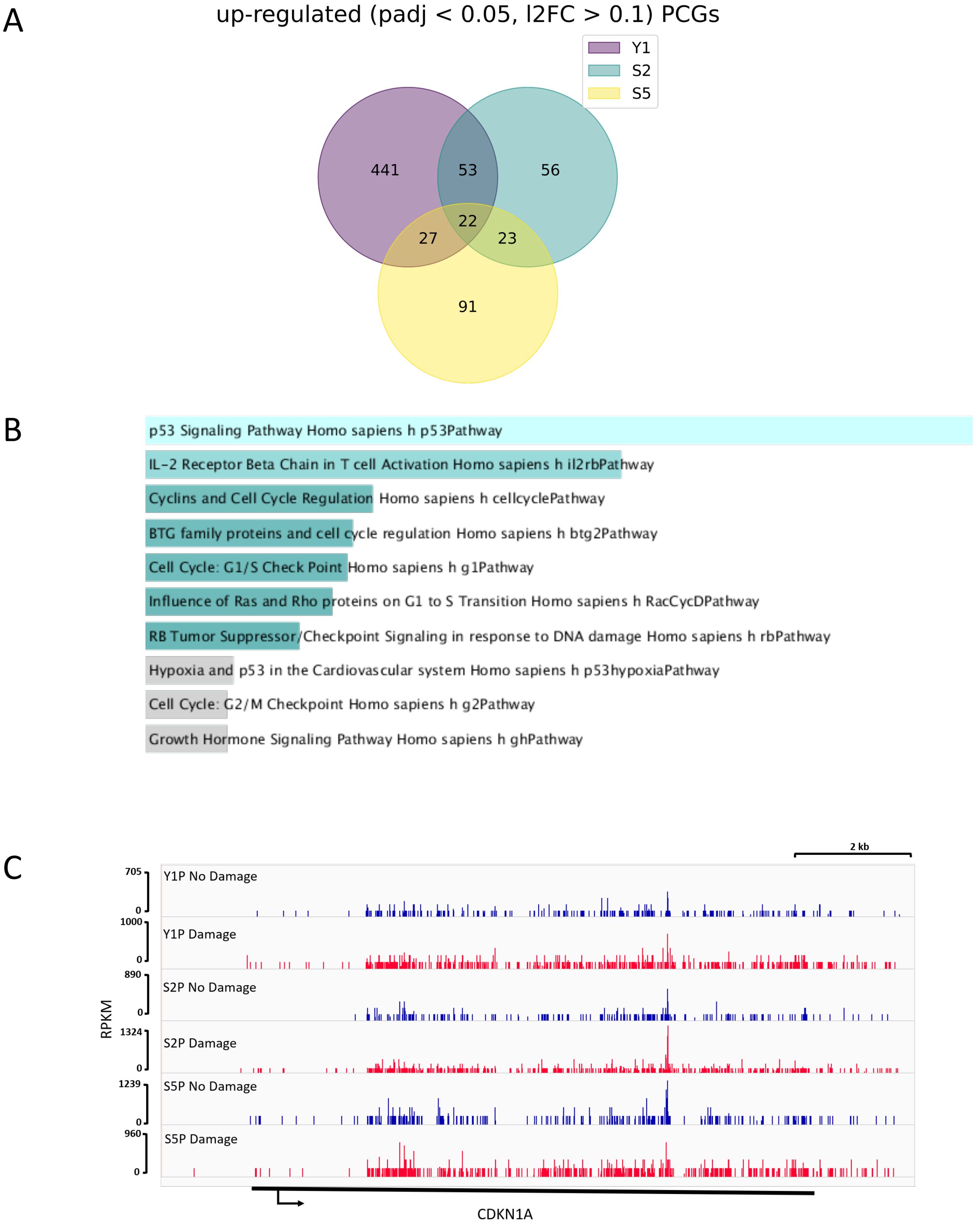

Figure S2. Analysis of up-regulated protein-coding genes upon DNA damage. **A)** Venn diagram of significantly up-regulated (padj < 0.05, $log_2FC > 0.1$) protein-coding genes based on read coverage across gene body from Y1P, S2P and S5P sample2s after IR**. B)** GO enrichment of significantly up-regulated genes across Y1P, S2P, S5P samples upon IR**. C)** IGV profile of mNET-seq signal across a representative up-regulated gene, CDKN1A**.**
