# Supplementary material for: Tyrosine 1–phosphorylated RNA polymerase II transcribes PROMPTs to facilitate proximal promoter pausing and induce global transcriptional repression in response to DNA damage
Source: Genome Res. 2024 Feb;34(2):201–16. doi: 10.1101/gr.278644.123 (PMC10984383; doi:10.1101/gr.278644.123)
Supplement: Supplement 3 [file Supplemental_Fig_S3.pdf]

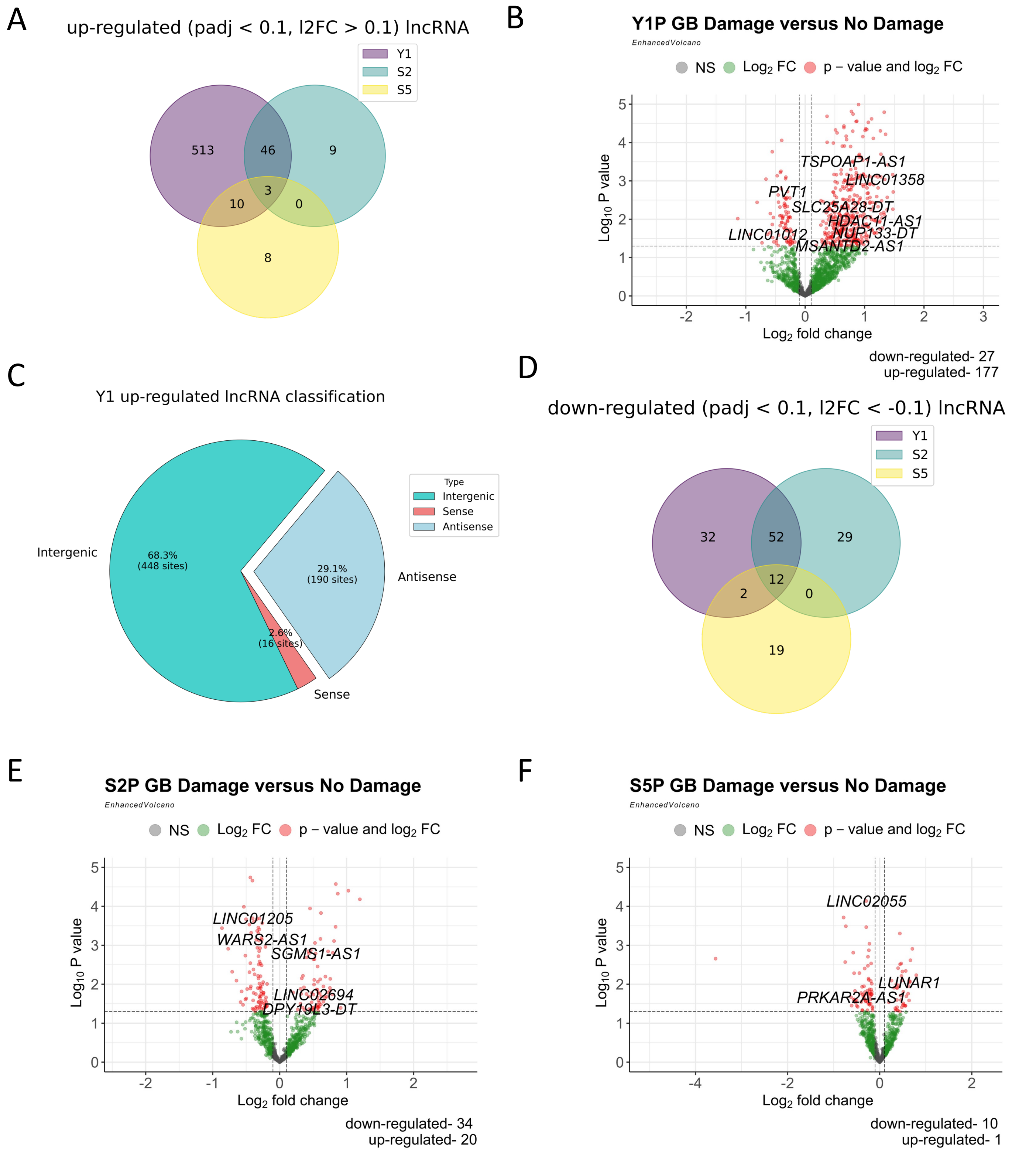

Figure S3. Analysis of long non-coding RNA (lncRNA) expression upon DNA damage. **A)** Venn diagram of significantly up-regulated (padj < 0.05, $log_2FC > 0.1$) lncRNA based on read coverage across gene body from Y1P, S2P and S5P samples after IR. **B)** Volcano plots of differentially expressed lncRNA based on read coverage across GB from Y1P samples upon IR**. C)** Pie chart showing proportion of differentially expressed (padj < 0.05) lncRNA which are sense, anti-sense and intergenic from Y1P samples upon IR. **D)** Venn diagram of significantly down-regulated (padj < 0.05, $log_2FC < -0.1$) lncRNA based on read coverage across gene body from Y1P, S2P and S5P samples after IR**. E)** Volcano plots of lncRNA based on read coverage across GB from S2P samples upon IR**. F)** Volcano plots of differentially expressed lncRNA based on read coverage across GB from S5P samples upon IR**.**
