# Supplementary material for: Tyrosine 1–phosphorylated RNA polymerase II transcribes PROMPTs to facilitate proximal promoter pausing and induce global transcriptional repression in response to DNA damage
Source: Genome Res. 2024 Feb;34(2):201–16. doi: 10.1101/gr.278644.123 (PMC10984383; doi:10.1101/gr.278644.123)
Supplement: Supplement 4 [file Supplemental_Fig_S4.pdf]

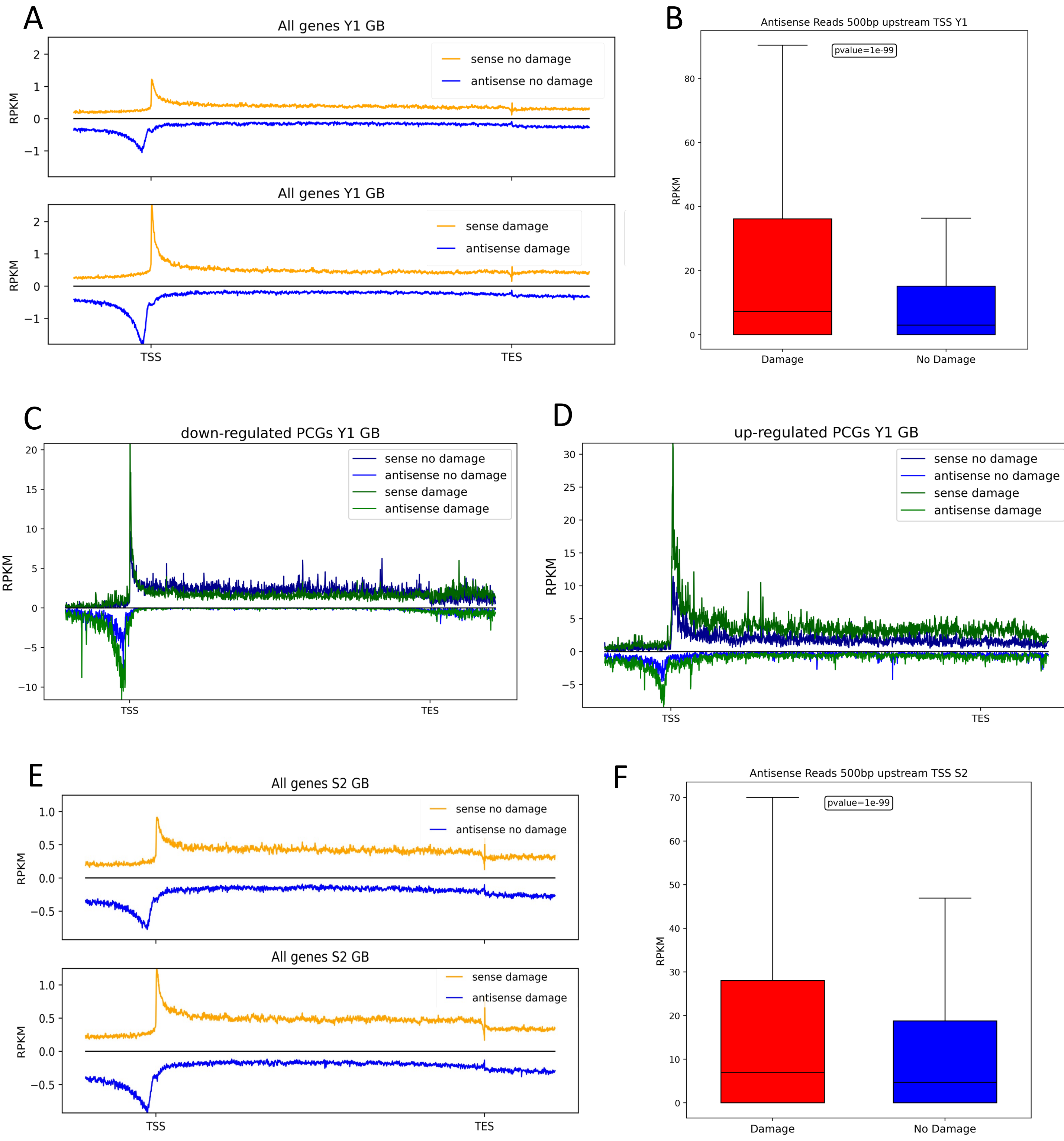

Figure S4. Analysis of PROMPTs genome wide in response to DNA damage**. A)** mNET-Seq metagene profiles showing sense and antisense transcription across GB of all genes from Y1P damage and no damage samples**. B)** Box plot indicates mNET-Seq read coverage across PROMPTs of all genes from Y1P damage and no damage samples**. C)** mNET-Seq metagene profiles showing sense and antisense transcription across GB of down-regulated genes from Y1P damage and no damage samples. **D)** mNET-Seq metagene profiles showing sense and antisense transcription across GB of up-regulated genes from Y1P damage and no damage samples. **E)** mNET-Seq metagene profiles showing sense and antisense transcription across GB of all genes from S2P damage and no damage samples**. F)** Box plot indicates mNET-Seq read coverage across PROMPTs of all genes from S2P damage and no damage samples. Two sample Wilcoxon test is used to compute statistical significance of PROMPT expression between damage and no damage sample.
