# Supplementary material for: Tyrosine 1–phosphorylated RNA polymerase II transcribes PROMPTs to facilitate proximal promoter pausing and induce global transcriptional repression in response to DNA damage
Source: Genome Res. 2024 Feb;34(2):201–16. doi: 10.1101/gr.278644.123 (PMC10984383; doi:10.1101/gr.278644.123)
Supplement: Supplement 5 [file Supplemental_Fig_S5.pdf]

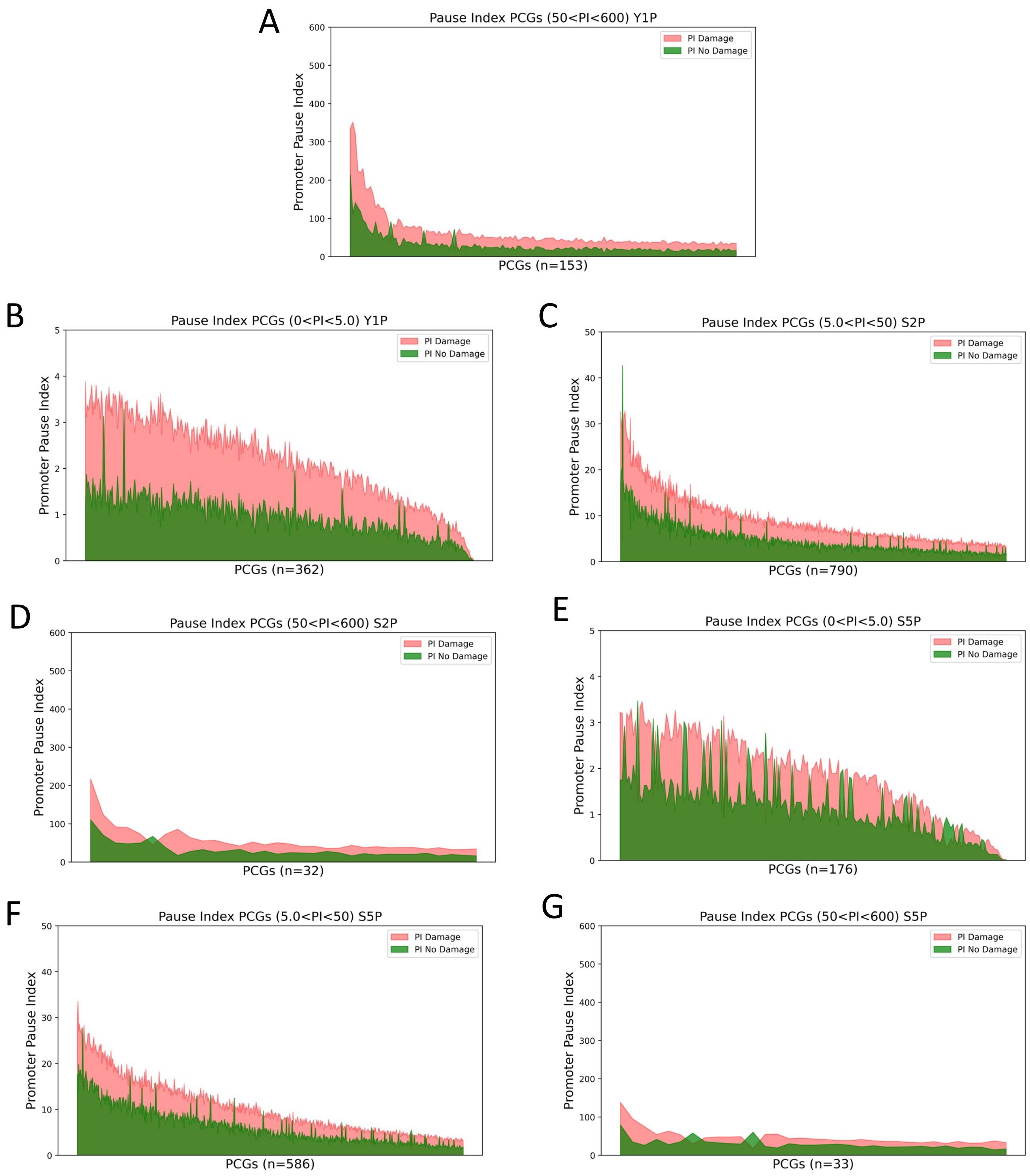

Figure S5. Analysis of promoter pausing index upon DNA damage. **A)** Line plot showing change in Pause Index(PI) of protein-coding genes belonging to high PI category (50 < PI < 600) from Y1P samples upon IR. **B)** Line plot showing change in Pause Index(PI) of protein-coding genes belonging to low PI category (0 < PI < 5) from S2P samples upon IR. **C)** Line plot showing change in Pause Index(PI) of protein-coding genes belonging to moderate PI category (5 < PI < 50) from S2P samples upon IR. **D)** Line plot showing change in Pause Index(PI) of protein-coding genes belonging to high PI category from S2P samples upon IR. **E)** Line plot showing change in Pause Index(PI) of protein-coding genes belonging to low PI category from S5P samples upon IR. **F)** Line plot showing change in Pause Index(PI) of protein-coding genes belonging to moderate PI category from S5P samples upon IR. **G)** Line plot showing change in Pause Index(PI) of protein-coding genes belonging to high PI category from S5P samples upon IR. Only genes that show significant (p<0.05) change in PI are plotted.
