# Supplementary material for: Tyrosine 1–phosphorylated RNA polymerase II transcribes PROMPTs to facilitate proximal promoter pausing and induce global transcriptional repression in response to DNA damage
Source: Genome Res. 2024 Feb;34(2):201–16. doi: 10.1101/gr.278644.123 (PMC10984383; doi:10.1101/gr.278644.123)
Supplement: Supplement 6 [file Supplemental_Fig_S6.pdf]

A

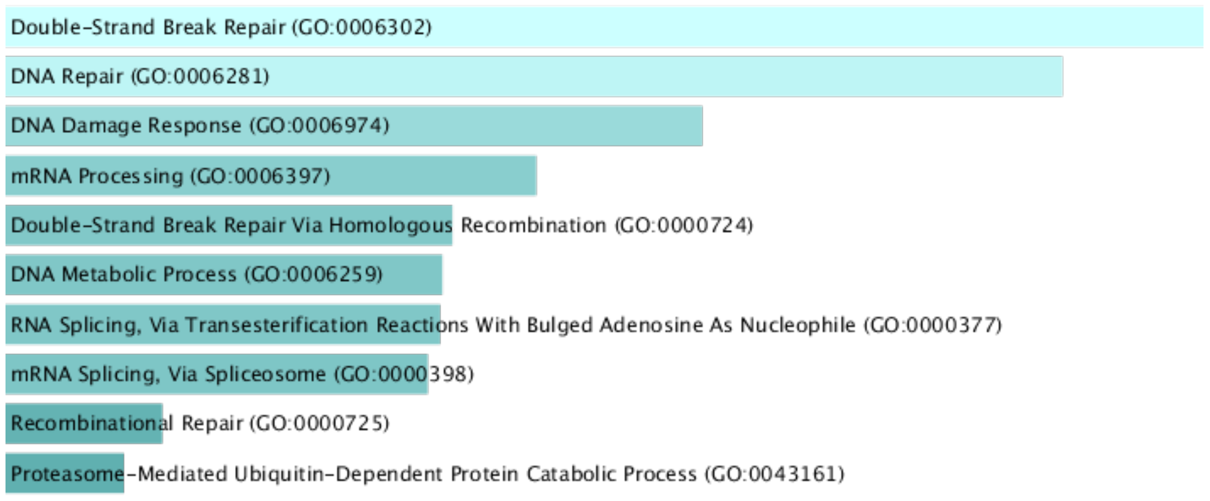

B

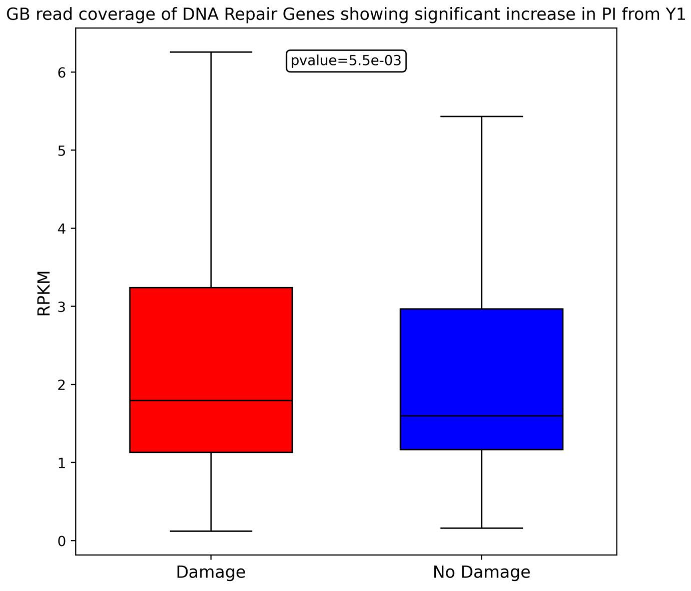

C

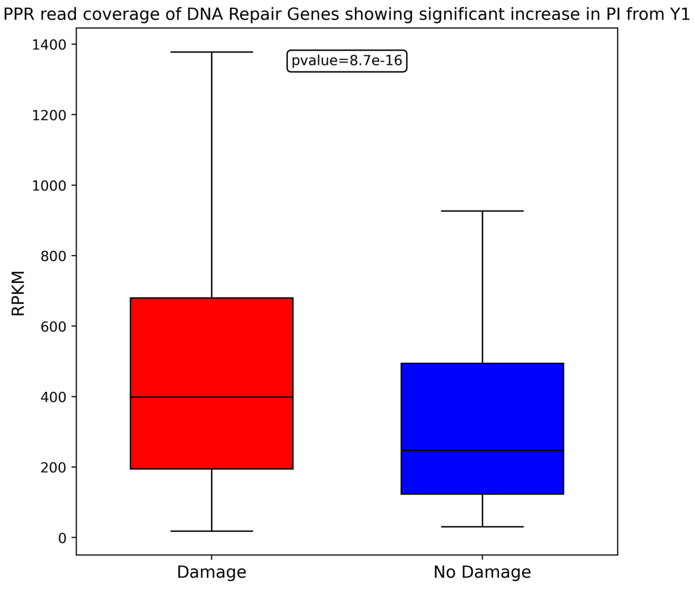

Figure S6. Enrichment analysis of genes with increased promoter pausing index upon DNA damage**. A)** GO enrichment of protein-coding genes showing significant increase in paise index from Y1P samples upon IR**. B)** Box plot indicates mNET-Seq read coverage across GB of DNA repair genes. **C)** Box plot indicates mNET-Seq read coverage across proximal promoter region of DNA repair genes. Two sample Wilcoxon test is used to compute statistical significance of coverage between damage and no damage samples.
