# Supplementary material for: Tyrosine 1–phosphorylated RNA polymerase II transcribes PROMPTs to facilitate proximal promoter pausing and induce global transcriptional repression in response to DNA damage
Source: Genome Res. 2024 Feb;34(2):201–16. doi: 10.1101/gr.278644.123 (PMC10984383; doi:10.1101/gr.278644.123)
Supplement: Supplement 7 [file Supplemental_Fig_S7.pdf]

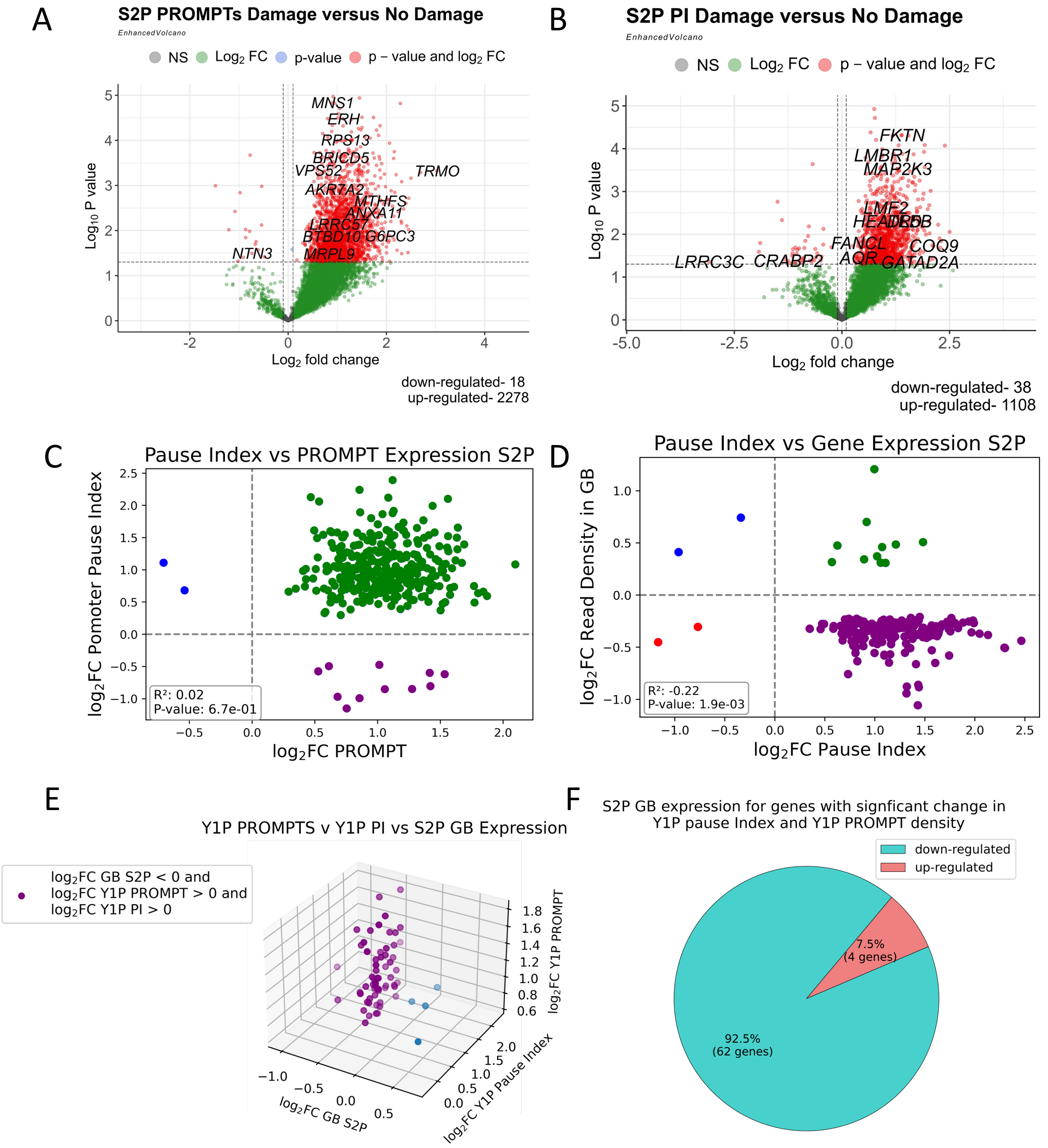

Figure S7. PROMPTs analysis of S2P RNAPII upon DNA damage. **A)** Volcano plots of differentially expressed PROMPTs from S2P upon IR. **B)** Volcano plots showing differential PI of protein-coding genes from S2P samples upon IR. **C)** Scatter plot showing log2FoldChange in PROMPT expression with associated log_2FoldChange in PI of protein-coding genes from S2P sample on radiation treatment**. D)** Scatter plot showing log2FoldChange in PROMPT expression with associated log2FoldChange in PI of protein-coding genes from S5P sample on radiation treatment. **E)** Three-dimensional scatter plot comparing log_2FoldChange in S2P GB expression with log_2FoldChange in Y1P PI and log2FoldChange in Y1P PROMPT of protein-coding genes from S2P samples upon IR treatment. Only protein-coding genes that show significant ($p < 0.05$) change in Y1P PROMPT expression, Y1P PI and S2P GB expression upon IR were included in the analysis**. F)** Pie chart showing percentage of down-regulated and up-regulated genes among the protein-coding genes that show significant ($p < 0.05$) change in Y1P PROMPT expression, Y1P PI and S2P GB expression upon IR.
