# Supplementary material for: Tyrosine 1–phosphorylated RNA polymerase II transcribes PROMPTs to facilitate proximal promoter pausing and induce global transcriptional repression in response to DNA damage
Source: Genome Res. 2024 Feb;34(2):201–16. doi: 10.1101/gr.278644.123 (PMC10984383; doi:10.1101/gr.278644.123)
Supplement: Supplement 8 [file Supplemental_Fig_S8.pdf]

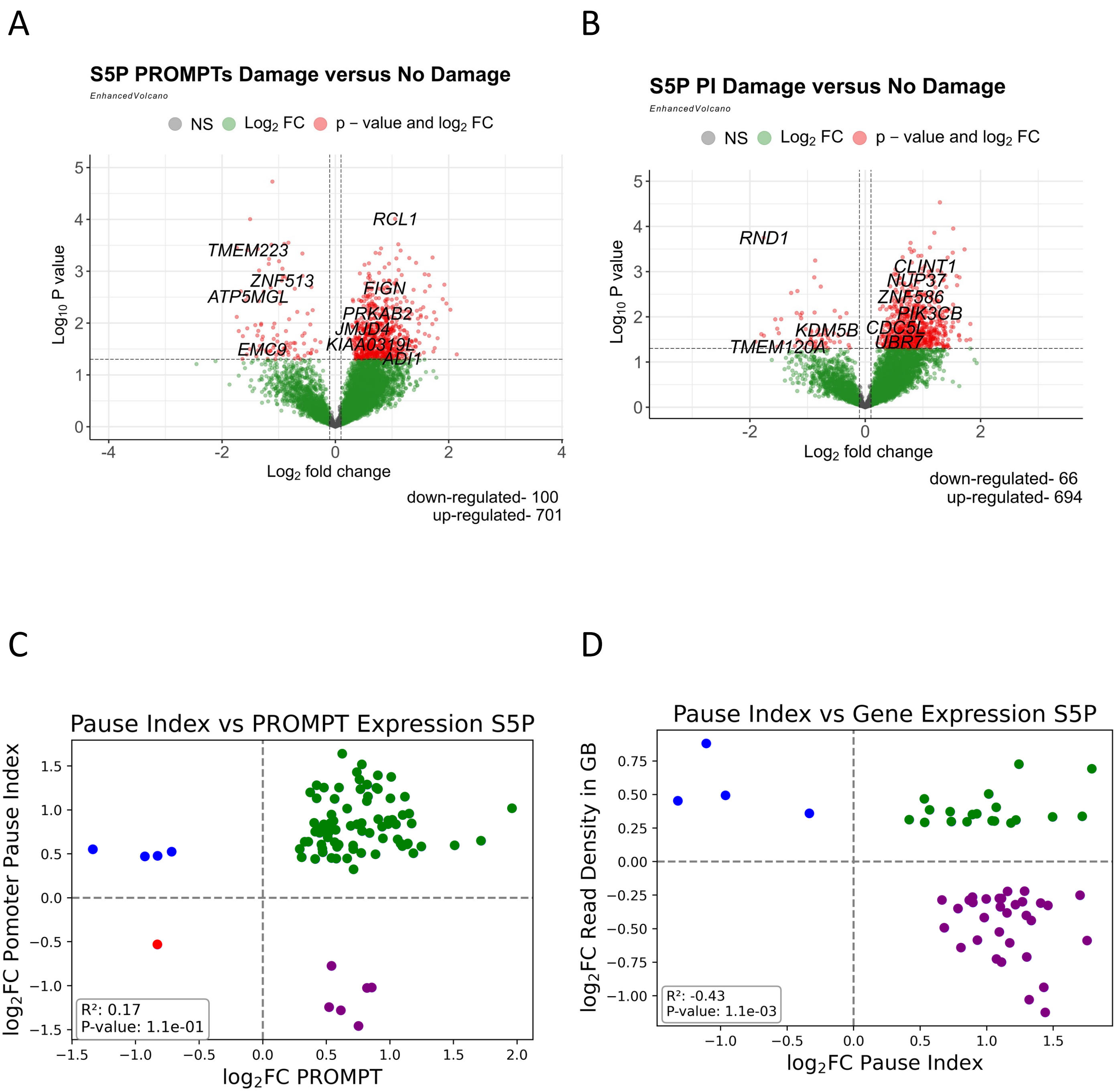

Figure S8. Promoter pausing index analysis of S5P RNAPII upon DNA damage. **A)** Volcano plots showing differential PROMPTs of protein-coding genes from S5P samples upon IR. **B)** Volcano plots showing differential PI of protein-coding genes from S5P samples upon IR. **C)** Scatter plot showing log_2FoldChange in PROMPTs expression with associated log_2FoldChange in PI of protein-coding genes from S5P sample on IR treatment. **D)** Scatter plot showing log_2FoldChange in GB expression with associated log_2FoldChange in PI of protein-coding genes from S5P sample upon IR treatment.
