# Supplementary material for: Tyrosine 1–phosphorylated RNA polymerase II transcribes PROMPTs to facilitate proximal promoter pausing and induce global transcriptional repression in response to DNA damage
Source: Genome Res. 2024 Feb;34(2):201–16. doi: 10.1101/gr.278644.123 (PMC10984383; doi:10.1101/gr.278644.123)
Supplement: Supplement 9 [file Supplemental_Fig_S9.pdf]

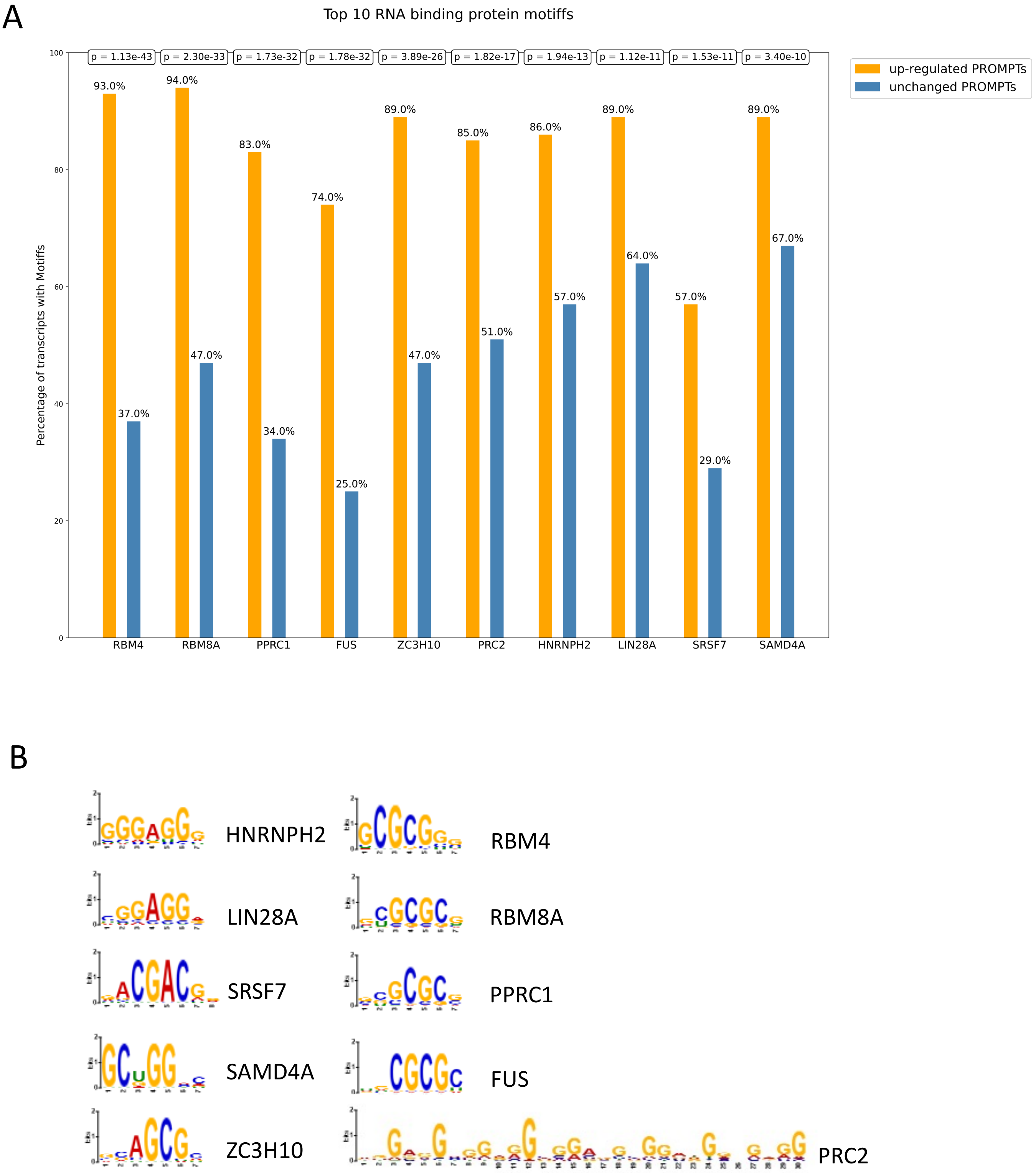

Figure S9. Enrichment analysis of top 10 RNA binding motifs on up-regulated PROMPTs vs unchanged PROMPTs. **A)** Bar charts showing percentage of sequences containing RNA binding protein motifs among the up-regulated (p value < 0.05, $\log_2 FC > 0.1$) PROMPTs which are correlated with down-regulated GB expression and increased PI upon IR from Y1P samples vs unchanged PROMPTs. Mann-Whitney *U* test was to compute statistical significance in the difference in enrichment of top 10 RNA binding protein motifs between up-regulated and unchanged PROMPTs**. B)** Binding motifs of 10 top RNA binging proteins ranked based on significant enrichment of binding sites on up-regulated PROMPTs compared with unchanged PROMPTs.
