# Supplementary material for: Tyrosine 1–phosphorylated RNA polymerase II transcribes PROMPTs to facilitate proximal promoter pausing and induce global transcriptional repression in response to DNA damage
Source: Genome Res. 2024 Feb;34(2):201–16. doi: 10.1101/gr.278644.123 (PMC10984383; doi:10.1101/gr.278644.123)
Supplement: Supplement 10 [file Supplemental_Fig_S10.pdf]

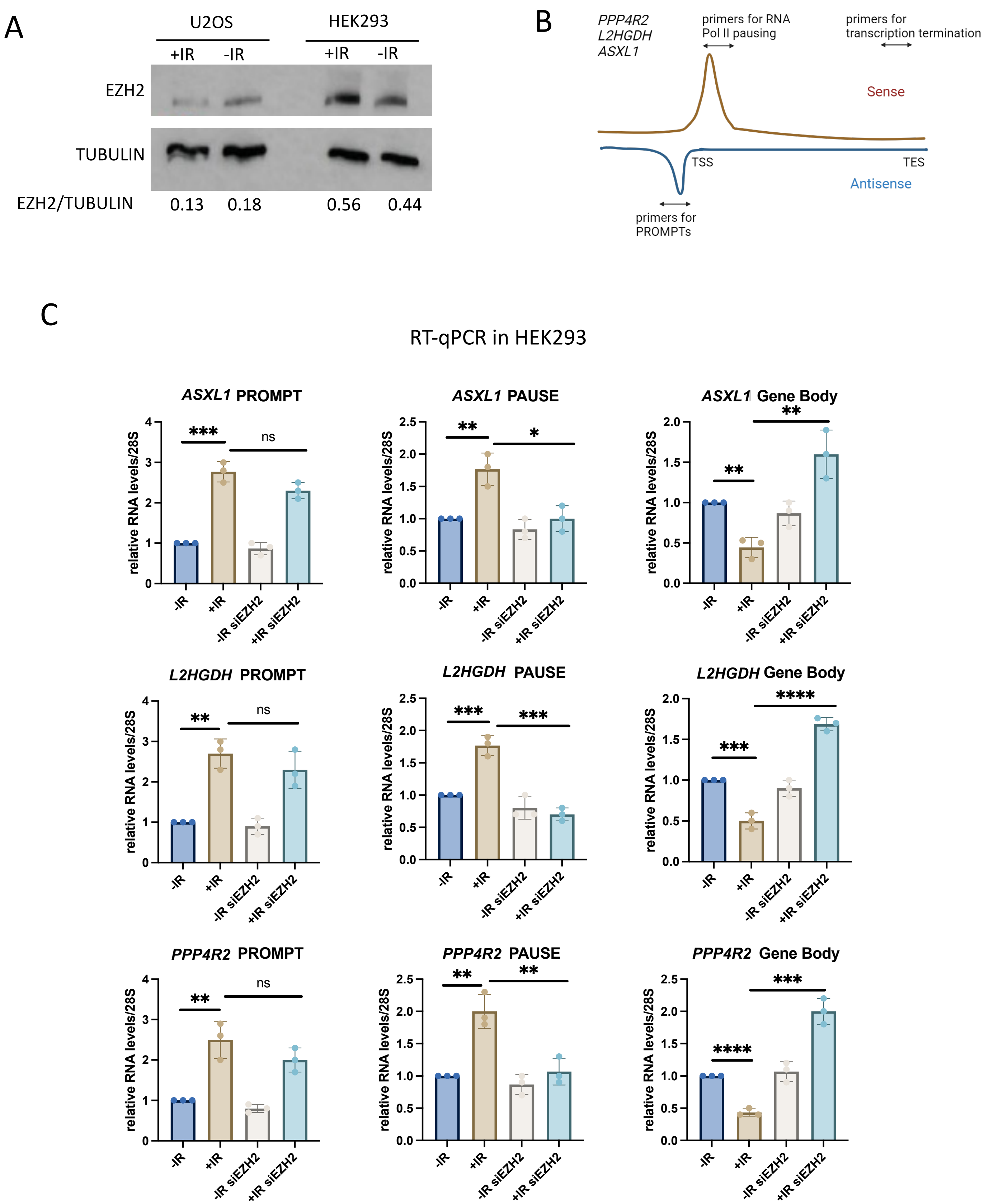

Figure S10. Analysis of protein and RNA levels. **A)** Western blot showing levels of EZH2 in U2OS and HEK293 cells upon IR. B) Positions of primers used for RT-qPCRs. C) Bar charts showing RT-qPCR analysis of PROMPTs, PAUSE and Gene Body derived RNA at three selected genes. Unpaired *t*-test was used to assess statistical significance. * < 0.05, ** < 0.01, *** < 0.001, **** < 0.0001.
