# Supplementary material for: Tyrosine 1–phosphorylated RNA polymerase II transcribes PROMPTs to facilitate proximal promoter pausing and induce global transcriptional repression in response to DNA damage
Source: Genome Res. 2024 Feb;34(2):201–16. doi: 10.1101/gr.278644.123 (PMC10984383; doi:10.1101/gr.278644.123)
Supplement: Supplement 11 [file Supplemental_Fig_S11.pdf]

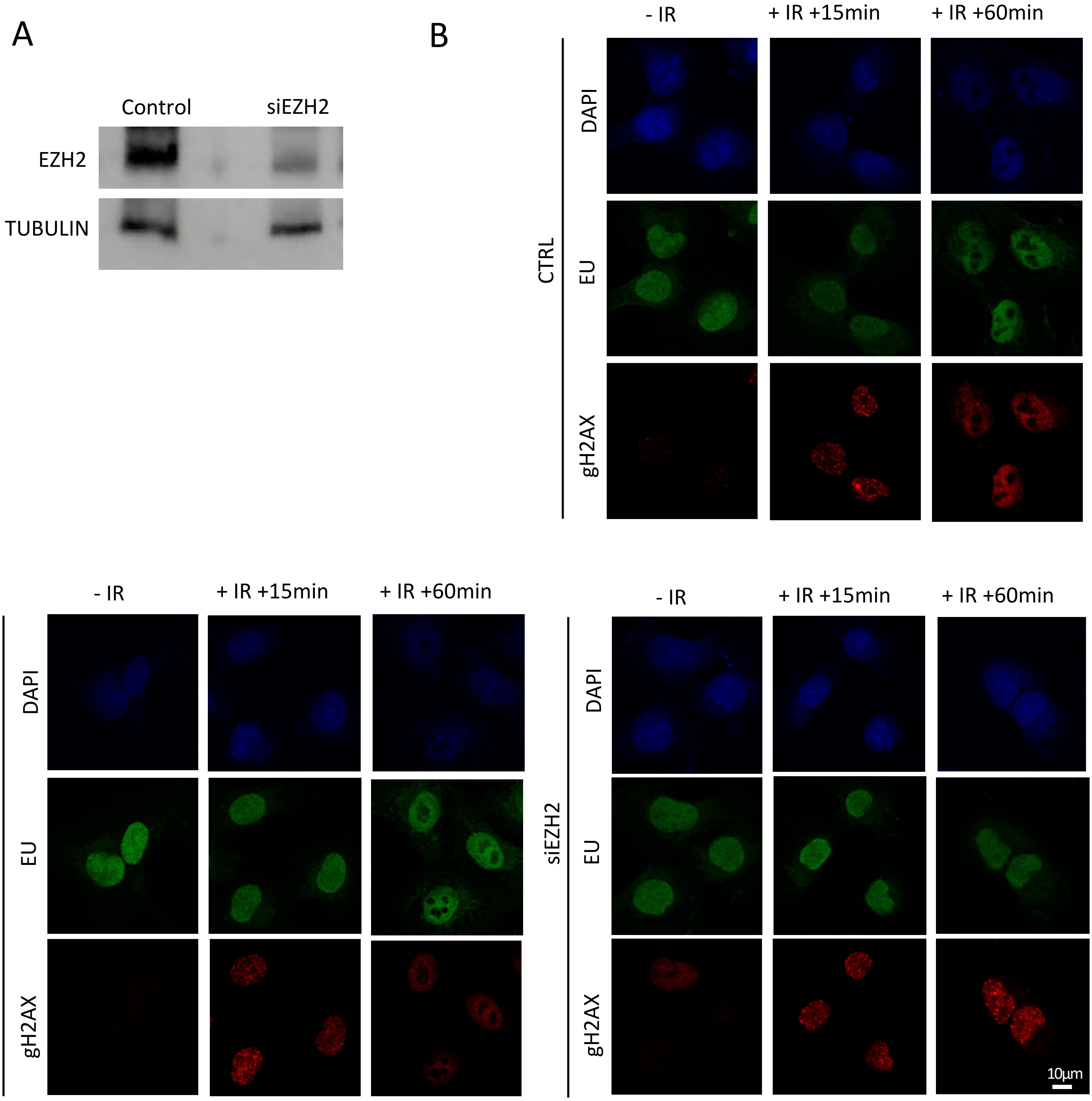

Figure S11. Analysis of nascent RNA levels using EU staining. **A)** Western blot showing levels of EZH2 in wt and siRNA treated cells. **B)** Representative images showing EU staining of nascent RNA in cells exposed to IR, DAPI stains nuclei, EU straining is shown in green and gH2AX is shown in red.
