# Supplementary material for: Tyrosine 1–phosphorylated RNA polymerase II transcribes PROMPTs to facilitate proximal promoter pausing and induce global transcriptional repression in response to DNA damage
Source: Genome Res. 2024 Feb;34(2):201–16. doi: 10.1101/gr.278644.123 (PMC10984383; doi:10.1101/gr.278644.123)
Supplement: Supplement 12 [file Supplemental_Fig_S12.pdf]

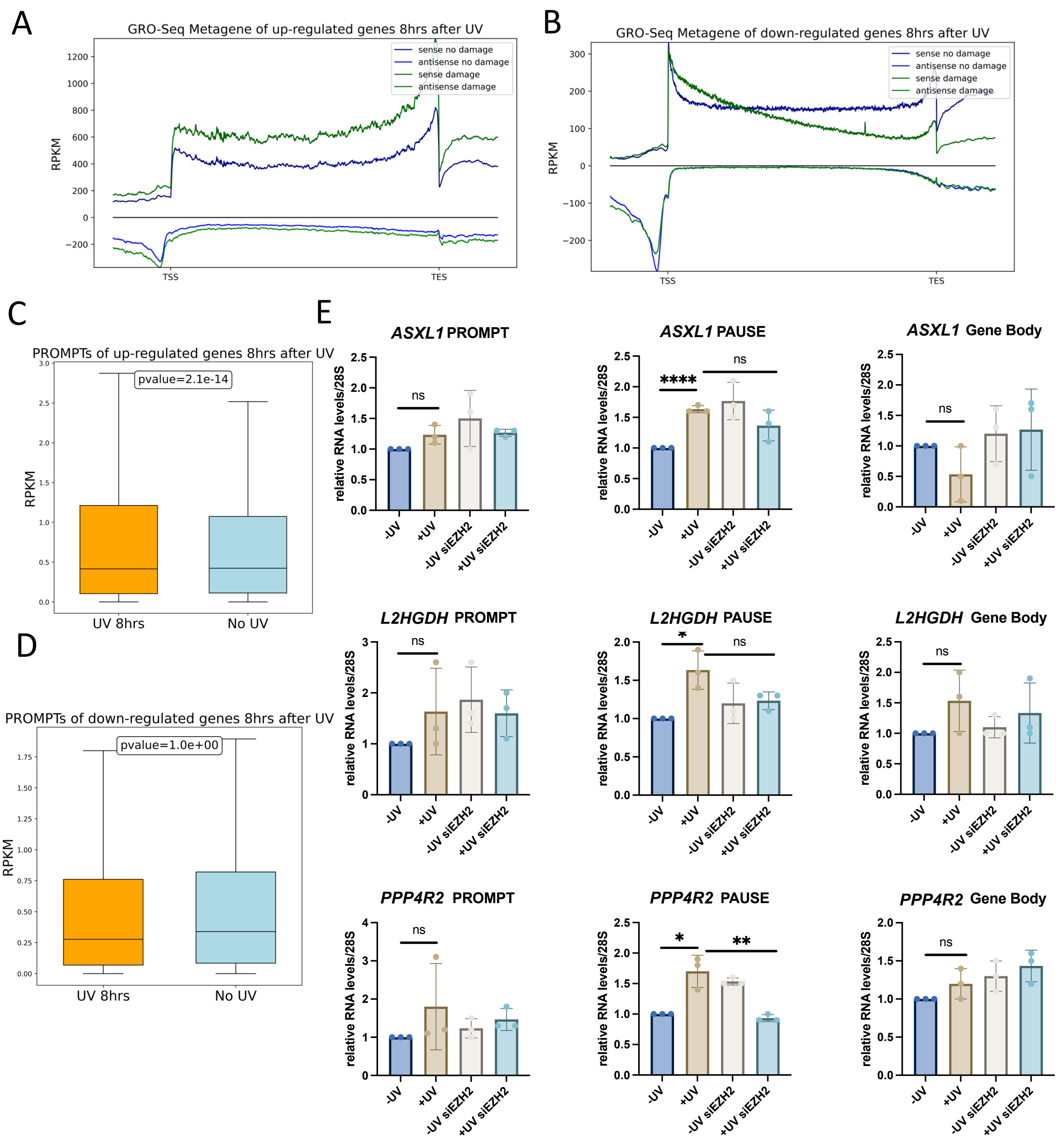

Figure S12. Analysis of nascent transcriptome following UV exposure. **A)** GRO-seq metagene profile showing sense and antisense transcription across GB of down-regulated genes from UV exposed and untreated samples**. B)** GRO-seq metagene profile showing sense and antisense transcription across GB of up-regulated genes from UV exposed and untreated samples**.** Differentially expressed genes were described as genes showing significant increase in gene expression based on RNA-Seq data comparing UV treated samples following 8hr incubation with untreated samples. **C)** Box plot indicates GRO-Seq read coverage across PROMPTs of up-regulated genes from UV exposed and untreated samples. Two sample Wilcoxon test is used to compute statistical significance of difference in PROMPT expression for down-regulated genes between from UV exposed and untreated samples**. D)** Box plot indicates GRO-Seq read coverage across PROMPTs of down-regulated genes from UV exposed and untreated samples. Two sample Wilcoxon test is used to compute statistical significance of difference in PROMPT expression for down-regulated genes between from UV exposed and untreated samples**. E)** Bar charts showing RT-qPCR analysis of PROMPTs, PAUSE and Gene Body derived RNA in U2OS cells following UV treatment at three selected genes. Unpaired *t*-test was used to assess statistical significance. * < 0.05.
