# Supplementary material for: Tyrosine 1–phosphorylated RNA polymerase II transcribes PROMPTs to facilitate proximal promoter pausing and induce global transcriptional repression in response to DNA damage
Source: Genome Res. 2024 Feb;34(2):201–16. doi: 10.1101/gr.278644.123 (PMC10984383; doi:10.1101/gr.278644.123)
Supplement: Supplement 13 [file Supplemental_Fig_S13.pdf]

ABL1 mRNA expression

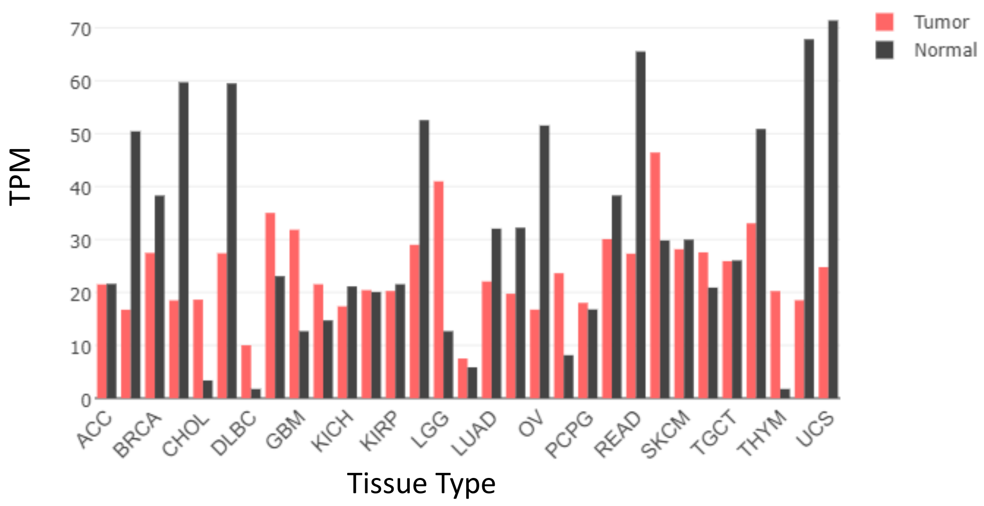

ACC – Adrenocortical carcinoma

BRCA – Breast Invasive

CHOL – Cholangio carcinoma

DLBC – Lymphoid Neoplasm Diffuse Large B-cell Lymphoma

GBM – Glioblastoma multiforme

KICH – Kidney Chromophobe

KIRP – Kidney renal papillary cell carcinoma

LGG – Brain Lower Grade Glioma

LUAD – Lung adenocarcinoma

OV – Ovarian serious cystadenocarcinoma

PCPG – Pheochromocytoma and Paraganglioma

READ – Rectum adenocarcinoma

SKCM – Skin Cutaneous melanoma

TGCT – Testicular Germ Cell Tumour

THYM – Thymoma

UCS – Uterine Carcinosarcoma

Figure S13. ABL1 expression in tumour vs non-tumour tissues. Transcripts Per Million (TPM) levels of ABL1 across various tissues comparing tumour expression. Data was procured from (http://gepia.cancer-pku.cn/about.html)
