# Supplementary material for: Tyrosine 1–phosphorylated RNA polymerase II transcribes PROMPTs to facilitate proximal promoter pausing and induce global transcriptional repression in response to DNA damage
Source: Genome Res. 2024 Feb;34(2):201–16. doi: 10.1101/gr.278644.123 (PMC10984383; doi:10.1101/gr.278644.123)
Supplement: Supplement 15 [file Supplemental_Table_S1.docx]

| **Reagent** | **Source** | **Identifier** |
| --- | --- | --- |
| **siRNA** | | |
| ON-TARGETplus siRNA EZH2 Human Smart pool | Dharmacon | L-004218-00 |
| ON-TARGETplus Non-targeting siRNA #1 | Dharmacon | D-001810-01 |
| **Chemicals** | | |
| 5-EU | Jena Bioscience | CLK-NOO2-10 |
| THPTA | Jena Bioscience | CLK-1010-100 |
| CuSO4 Pentahydrate | Sigma | C8027 |
| Sodium L-ascorbate | Sigma | A7631 |
| Aminoguanidine hydrochloride | Sigma | 396494 |
| Biotin-PEG_3_-azide | Cayman Chemicals | 23419 |
| Imatinib | ApexBio | B2171 |
| DAPI | Abcam | AB104139 |
| **Antibodies** | | |
| anti-Biotin | Rockland | 200-301-098S |
| anti-Ezh2 | Cell Signalling Technologies | 5246T |
| anti-γH2AX (phosphoS139) | Abcam | ab11174 |
| anti-RNAPII-CTD phospho-Ser2 | Abcam | ab5095 |
| anti-RNAPII-CTD phospho-Ser5 | Abcam | ab5131 |
| anti-RNAPII-CTD phospho-Tyr1 | Active Motif | [3D12], 61383 |
| anti beta tubulin | Abcam | ab6046 |
| Alexa Fluor 555 goat anti-rabbit | Invitrogen | A21428 |
| Alexa Fluor 488 goat anti-mouse | Invitrogen | A11001 |

Supplemental Table 1: List of resources used in this study.
