# Supplementary material for: Tyrosine 1–phosphorylated RNA polymerase II transcribes PROMPTs to facilitate proximal promoter pausing and induce global transcriptional repression in response to DNA damage
Source: Genome Res. 2024 Feb;34(2):201–16. doi: 10.1101/gr.278644.123 (PMC10984383; doi:10.1101/gr.278644.123)
Supplement: Supplement 16 [file Supplemental_Table_S2.docx]

| **Primer** | **Sequence (5’-3’)** |
| --- | --- |
| L2HGDH_IN_REV | GCACCAGTCAGAAGTGTCGG |
| L2HGDH_EX_REV | TCAAGACAGAGCCACCTGCT |
| L2HGDH_EX_FWD | GGCATTGTGGACTATCGGCA |
| ASXL1_IN_REV | AGTCCAAGAATCACTGCACCAA |
| ASXL1_EX_REV | AAAAGGCTGATTCGGCCAGG |
| ASXL1_EX_FWD | CCCTCTCGCATGCCTCAATG |
| USP34_IN_REV | TTCCGCCAGCAGAAAACTGT |
| USP34_EX_REV | TCCCGGAGCACTTGAACTTG |
| USP34_EX_FWD | GCAATGTCTATGCTGCTTCAAGG |
| 28S_FWD | TTCCCTCCGAAGTTTCCCTC |
| 28S_RWD | ACTAGGCACTCGCATTCCAC |
| PPP4R2_FWD_PRO_RT | GCGCATTCTGAACGTGAAGTGTA |
| PPP4R2_REV_PRO | GACTGGACGGTCAGGCGA |
| PPP4R2_FWD_PAUSE | ACGTACCGGGCGCCAT |
| PPP4R2_PAUSE_REVRT | GAAGCGTGCGAGAGAGCAAG |
| ASXL1_FWD_PRO_RT | GTCCTGAAGCAAAAAGCACCG |
| ASXL1_REV_PRO | GCGAGGTGCGAGAACTGG |
| ASXL1_FWD_PAUSE | CTCAGCAGAGCGGGAAAGC |
| ASXL1_PAUSE_REV_RT | ATCGCTAAGGAATGCGGCG |
| L2HGDH_PAUSE_REVRT | CCACACAGCGGTCTTGGC |
| L2HGDH_PAUSE_FWD | GCTGCGTTATTTGGTTGGTGC |
| L2HGDH_FWD_PRO | AAAACCTGGCCCTTTGGGTC |
| L2HGDH_REV_PRO_RT | CGTAATCCCGCCCAGAGTTT |
| FISH-PLA_PRO_FWD_1 | GTCCGCCTGCAACCCTCTCGCCTGACCGTCCAGTCTTCAGCTCCGCGGAGAAAAAAAAAAAAAAAAAAAAAAAAAAAAAAAAAAAAAAAATATGACAGAACTAGACAC |
| FISH-PLA_PRO_FWD_2 | TCACAGCAACTGCATAGGGCATCAGTACCAGTGCCCTTACTGTACAGATGAAAAAAAAAAAAAAAAAAAAAAAAAAAAAAAAAAAAAAAATATGACAGAACTAGACAC |
| FISH-PLA_PRO_FWD_3 | AGTGTTGGGATCACCGGCCTGAGCCCCCTGCCCGGCCTCGCTCTACGTTTAAAAAAAAAAAAAAAAAAAAAAAAAAAAAAAAAAAAAAAATATGACAGAACTAGACAC |

Supplemental Table 2: List of oligonucleotides used in this study.
